# Trends of obesity management in adults: an analysis across guidelines in China and in Europe

**DOI:** 10.1093/pcmedi/pbaf018

**Published:** 2025-07-22

**Authors:** Shen Qu, Le Bu, Jiajun Zhao

**Affiliations:** Department of Endocrinology and Metabolism, Shanghai Tenth People's Hospital, School of Medicine, Tongji University, Shanghai 200072, China; Department of Endocrinology and Metabolism, Shanghai Tenth People's Hospital, School of Medicine, Tongji University, Shanghai 200072, China; Department of Endocrinology, Shandong Provincial Hospital affiliated with Shandong University, Jinan 250021, China

## Introduction

Obesity is a multifaceted, chronic condition influenced by genetic, metabolic, psychological, and social factors, necessitating a comprehensive and personalized approach to treatment. Managing weight effectively remains a significant challenge, requiring sustained efforts over the long term. Both the public and healthcare professionals need to gain a deeper understanding of obesity, moving beyond its stigma [1]. In July 2024, the Chinese Society of Endocrinology released China's first “Guideline for Long-Term Weight Management and Clinical Practice of Anti-Obesity Medications” (the English version is provided in the online supplementary material). Two weeks later, the European Association for the Study of Obesity published “A New Framework for the Diagnosis, Staging, and Management of Obesity in Adults” [2]. Both guidelines underscored obesity as a chronic disease that demands new assessment metrics, early medical intervention, and long-term management. Due to the differing characteristics of Eastern and Western populations, there are variations in diagnostic criteria, classification, staging, and treatment strategies. This document aims to summarize the key points from both guidelines, as outlined in Table 1.

**Table 1:** An analysis across guidelines in China and in Europe.

Content	Guideline for Long-term Management and Clinical Application of Obesity Medications [1]	A New Framework for the Diagnosis, Staging, and Management of Obesity in Adults [2]
Definition	Obesity is a chronic, relapsing disease resulting from genetic and environmental factors and excessive accumulation of adipose tissue endangers health.	Obesity is a multifactorial, chronic, relapsing, non-communicable disease characterized by abnormal and/or increased appetite, body fat accumulation, and health risks.
Diagnosis	Comprehensive assessment based primarily on BMI combined with waist circumference, waist-to-hip ratio, body fat percentage, visceral fat	Emphasizing the accumulation of abdominal fat and health impacts, it is recommended to use waist-to-height ratio as a diagnostic criterion
Classification	Classify according to cause and fat distribution, such as simple obesity and secondary obesity According to body fat distribution, it is divided into abdominal obesity and systemic obesity Classification based on artificial intelligence algorithm: metabolically healthy obese, hypermetabolic obesity-hyperuricemia subtype, hypermetabolic obesity-hyperinsulinemia subtype, and hypometabolic obesity	ABCD classification, diagnosis of obesity based on identification of abnormalities and/or excessive fat accumulation (anthropometric component) and analysis of their current and potential impact on health (clinical assessment)
Stage	Not yet uniform and usually staged according to degree of obesity and complications	Staging according to clinical presentation and severity of complications
Treatment Goals	Achieve individualized optimal body weight, reduce the risk of complications, and improve metabolic parameters and quality of life	Obesity is managed and treated more broadly than weight-loss goals alone, including preventing, addressing, or improving obesity-related complications, improving quality of life and mental health, and improving physical/social functioning and health status
Treatment Measures	Lifestyle intervention, pharmacotherapy, and metabolic surgery	Emphasize personalized treatment options, lifestyle intervention, drug therapy, metabolic surgery, psychological intervention
AOM^a^ maintenance	It is recommended to maintain the original anti-obesity medications regimen and lifestyle interventions during the maintenance phase after intensive weight loss treatment.	Emphasis on the need for a long-term or lifelong comprehensive treatment plan. No specific recommendation for AOM.
Outcomes	Focus on improving long-term weight management and complications	Highlight prevention and improvement of obesity-related complications
Mechanism of exploration of obesity	Focus on endocrine and metabolic abnormalities on obesity	To investigate the relationship between obesity and metabolic and mental health

^a^AOM, Anti-obesity medication.

## Definition and diagnosis

Both guidelines have updated and refined the definition and diagnosis of obesity. They defined obesity as a chronic, relapsing disease characterized by excessive accumulation of adipose tissue that poses significant health risks, highlighting its complex etiology. While body mass index (BMI) remains the primary diagnostic criterion, it often fails to reflect the role of adipose tissue. As a result, the diagnosis of obesity now requires qualitative assessments and severity grading based on metabolic abnormalities and organ function impairment.

The European guideline emphasized a dual-faceted approach to obesity assessment, diagnosing obesity through the identification of abnormal and/or excessive fat accumulation, and evaluating its current and potential health impacts. Abdominal fat accumulation is particularly associated with an increased risk of cardiometabolic complications including type 2 diabetes [3]. It is a stronger determinant of disease progression than BMI, especially in individuals with BMI levels below the obesity threshold [4]. Meanwhile, the Chinese guideline advocated for using BMI, waist circumference, waist-to-hip ratio, body fat percentage, and visceral fat as key indicators for diagnosing obesity. These differing approaches highlight the complexity involved in defining the disease.

## Classification and staging

While a universally accepted standard for obesity classification and staging remains elusive, obesity can be categorized based on its etiology into primary and secondary forms, or by fat distribution into abdominal and generalized phenotypes. Research in China has suggested categorizing obesity into subtypes based on metabolic characteristics such as skin manifestations, glucose and lipid metabolism, inflammation levels, and fat distribution patterns [5, 6]. Recent studies utilizing artificial intelligence algorithms have proposed a novel metabolic subtyping of obesity [7, 8]. This approach identified four distinct subtypes: metabolically healthy obesity (MHO), high metabolic obesity with hyperuricemia (HMO-U), high metabolic obesity with hyperinsulinemia (HMO-I), and low metabolic obesity (LMO). Individuals with MHO exhibited a favorable metabolic profile and the lowest risk of complications; HMO-U was characterized by elevated uric acid levels; HMO-I by hyperinsulinemia (excessive insulin secretion); and LMO by relative insulin deficiency and hyperglycemia. This method has enhanced our understanding of the pathophysiological heterogeneity of obesity and enables clinicians to anticipate post-treatment metabolic responses based on subtype classification.

In contrast, the 2017 AACE/ACE guideline introduced the ABCD classification based on increased adiposity [9]. This system incorporated a multi-axial coding structure, encompassing: (A) etiological factors contributing to obesity; (B) BMI values; (C) obesity-related complications; and (D) the severity of these complications. The European guideline continued to use this classification.

## Obesity management

### Initial treatment

Both guidelines addressed the initial medical management of obesity, covering a range of interventions from lifestyle modifications to the initiation of pharmacotherapy. The indications for initial treatment should expand from obesity to overweight (if accompanied by metabolic abnormalities). Particularly for patients with a BMI ≥ 25 kg/m², a waist-to-height ratio > 0.5, and medical, functional, or psychological impairments or complications, anti-obesity medications should be considered, regardless of BMI.

The European guideline emphasized three key principles for initial treatment: individualized approach, shared decision-making, and dynamic treatment adaptation. The Chinese guideline advocated a strategy of: early intervention, early pharmacotherapy, and early combination therapy, which included lifestyle modifications and, when necessary, early pharmacological intervention, along with combining multiple treatment methods to enhance treatment efficacy and patient adherence.

### Long-term management

Both guidelines stressed the importance of long-term management, even life-long treatment, of obesity. The European guideline specified that the objectives of obesity management should extend beyond simple weight reduction to include the prevention, resolution, or improvement of obesity-related comorbidities.

The Chinese guideline recommended a structured approach to long-term weight management, which consists of an intensive treatment phase followed by a maintenance phase. Lifestyle interventions served as the cornerstone of long-term weight management. Regarding pharmacological management, the guideline suggested early use of anti-obesity medications when lifestyle interventions are insufficient.

Advancements in pharmaceutical development have established anti-obesity medications as a vital treatment modality for long-term weight management, especially given the improved efficacy and safety profiles of newer agents. Furthermore, the guidelines highlighted the critical role of long-term pharmacotherapy in achieving and maintaining weight loss, thereby mitigating weight regain—a known challenge in long-term weight management. It is recommended to continue the original anti-obesity medication regimen and lifestyle interventions during the maintenance phase following intensive weight-loss treatment. The European guideline stresses the need for a long-term or even lifelong comprehensive treatment plan, although it does not provide specific recommendations for anti-obesity medications.

Currently available anti-obesity medications in China include weekly injection formulations such as semaglutide and tirzepatide. Notably, the dual Gastric inhibitory polypeptide/glucagon-like peptide 1 (GIP/GLP-1) receptor agonism of tirzepatide has been associated with greater weight reduction compared to the GLP-1 receptor agonist semaglutide in clinical trials [10].

## Treatment goals

The overarching goal is to achieve long-term, sustainable weight management and the associated health benefits. The Chinese guideline advocated for establishing phased, specific weight-loss targets to facilitate progress and enhance adherence. Upon reaching the initial target, subsequent goals can be set, ultimately guiding the individual toward their personalized optimal weight. The European guideline recommended that adult obesity management should consider individualized treatment goals. Compared to the “take it one step at a time” approach recommended by the European guideline, the “step by step” strategy outlined in the Chinese guideline provided greater precision and clarity, potentially offering advantages in clinical practice.

## Multidisciplinary model of obesity management

Both guidelines emphasized the importance of a multidisciplinary approach to obesity management. Obesity clinical centers in China have evolved beyond the traditional multidisciplinary team model by implementing centralized management and personalized care. Following this assessment, an optimal, personalized weight-management plan is developed, incorporating appropriate lifestyle and pharmacological interventions. This comprehensive, multidisciplinary management and personalized treatment strategy aims to optimize treatment outcomes and maximize long-term health benefits for individuals with obesity.

## Summary

Obesity is a chronic, relapsing condition characterized by a level of complexity greater than that of many other chronic diseases. Because of this complexity, a significant translational gap remains between theoretical understanding and clinical practice in the diagnosis, assessment, staging, and management of obesity. This disparity highlights the urgent need for standardized, evidence-based treatment and management protocols. Effective obesity management requires a comprehensive, holistic approach that goes beyond simple weight reduction, taking into account its multifaceted impact on various physiological systems, including metabolic, endocrine, and psychological health. In clinical research, therapeutic strategies for obesity should not be limited to isolated pharmacological or surgical interventions. The judicious integration of pharmacotherapy with evidence-based lifestyle modifications can significantly improve metabolic profiles and overall health outcomes. This comprehensive, integrated approach is crucial for effectively addressing the obesity epidemic and managing obesity and its associated comorbidities.

## Supplementary Material

pbaf018_Supplemental_File
