# Supplementary material for: Trends of obesity management in adults: an analysis across guidelines in China and in Europe
Source: Precis Clin Med. 2025 Jul 22;8(4):pbaf018. doi: 10.1093/pcmedi/pbaf018 (PMC12527333; doi:10.1093/pcmedi/pbaf018)
Supplement: pbaf018_Supplemental_File [file pbaf018_supplemental_file.docx]

**Guideline for chronic weight management and clinical practice of anti-obesity medications (2024 version)**

Chinese Society of Endocrinology (CSE)

**Abstract**

Novel anti-obesity medications (AOMs) are being introduced in the context of rapid growth of obesity globally. In particular, the new generation of incretin-based therapy has made a breakthrough for the weight management in patients with obesity, with exceptional weight reduction, comprehensive cardiometabolic benefits, as well as favorable safety profile. However, until now, there is still lack of standardized clinical care with novel AOMs. Given the chronic and recurrent nature of obesity, long-term weight management is especially important. Despite this, we have not established a standard of the chronic weight management in obesity. To address this, the steering board invited experts with multidisciplinary expertise in obesity management to update the standard of care in obesity management, incorporating the concept of chronic weight management and extensive clinical experience with AOMs. It is expected that this guideline will provide comprehensive clinical guidance for weight management and long-term benefits to the patients with obesity.

**Keywords:** obesity; weight management; chronic; anti-obesity medication; guideline

Contents

[1. Overview of obesity 4](#_Toc203118760)

[1.1 Definition and epidemiology of obesity 4](#_Toc203118761)

[1.2 Obesity-related comorbidities and their harms 5](#_Toc203118762)

[1.3 Definition and diagnosis of obesity 6](#_Toc203118763)

[1.4 Classification and staging of obesity 7](#_Toc203118764)

[2. General principles for chronic management and diagnosis and treatment pathways for obesity 8](#_Toc203118765)

[2.1 Principles of chronic obesity management 8](#_Toc203118766)

[2.2 Patient diagnosis and treatment pathways 9](#_Toc203118767)

[2.2.1 Patient identification and initial assessment 9](#_Toc203118768)

[2.2.2 Whole-body assessment 10](#_Toc203118769)

[2.2.3 Comprehensive diagnosis of obesity 11](#_Toc203118770)

[2.2.4 Motivational interviewing 11](#_Toc203118771)

[2.2.5 Weight-loss goals 12](#_Toc203118772)

[2.3 The overall principles of the implementation of weight-loss strategies and long-term maintenance 13](#_Toc203118773)

[3. Lifestyle interventions in long-term weight management 15](#_Toc203118774)

[3.1 Diet management 15](#_Toc203118775)

[3.2 Exercise intervention 17](#_Toc203118776)

[3.3 Behavior modification 17](#_Toc203118777)

[3.4 Psychosocial support 18](#_Toc203118778)

[4. Weight-loss drug therapy in long-term weight management 18](#_Toc203118779)

[4.1 Overview of the development of weight-loss drugs 19](#_Toc203118780)

[4.2 Introduction to the classification of weight-loss drugs 21](#_Toc203118781)

[4.2.1 Lipase inhibitors 21](#_Toc203118782)

[4.2.2 NuSH receptor agonists 22](#_Toc203118783)

[4.3 Extra-weight loss benefits of NuSH receptor agonists 31](#_Toc203118784)

[4.4 Adverse reactions of NuSH receptor agonists and therapeutic regimen 32](#_Toc203118785)

[4.5 Monitoring and adjustment of weight-loss drugs in long-term management 34](#_Toc203118786)

[4.6 Long-term medication for weight maintenance after weight loss 34](#_Toc203118787)

[4.7 Combined application of weight-loss drugs 36](#_Toc203118788)

[5. Medications for common comorbidities of obesity 37](#_Toc203118789)

[6. Treatment of special populations 41](#_Toc203118790)

[6.1 Childhood Obesity 41](#_Toc203118791)

[6.2 Gestational obesity 42](#_Toc203118792)

[6.3 The elderly 43](#_Toc203118793)

[6.4 Patients with liver or kidney insufficiency 44](#_Toc203118794)

[6.5 Perioperative of metabolic surgery 45](#_Toc203118795)

[6.6 Obesity syndrome 46](#_Toc203118796)

# 1. Overview of obesity

## 1.1 Definition and epidemiology of obesity

Obesity is a chronic, progressive, relapsing disease caused by a combination of genetic and environmental factors with excessive accumulation or abnormal distribution of adipose tissue. According to the 2024 World Obesity Atlas, 42% of the adults (about 2.2 billion) were overweight or obese in 2020, and 54% (3.3 billion) are expected to be overweight or obesity by 2035 globally^[1]^. The data from China indicated that the prevalence of overweight and obesity reached 50.7% (comprising 34.3% overweight and 16.4% obese) according to the national diagnostic criteria (overweight defined as a body mass index (BMI) of 24-27.9 kg/m^2^, obesity defined as a BMI ≥28 kg/m^2^) in 2018. This prevalence has tripled since 2004 and is expected to reach 70.5% (610 million) by 2030^[2]^. Among children under 6 years, the prevalence of overweight and obesity is 6.8% and 3.6%, respectively, while among children and adolescents aged 6-17 years, the figures are 11.1% and 7.9%^[3]^. In addition, the prevalence of abdominal obesity (waist circumference ≥ 90 cm in men and ≥80 cm in women) among individuals with normal BMI in China is concerning, at 9.1% for men and 14.3% for women, reflecting a 3-4 fold increase since 1993^[4]^.

Lifestyles change including diet, nutrition, exercise, and socio-economic patterns are the main drivers of increased overall prevalence. The "carbohydrate-insulin model" explains this trend how high-carbohydrate, processed foods contribute to energy imbalance, leading to increased fat storage, heightened hunger, and reduced energy expenditure^[5]^. Factors such as rural versus urban living, lifestyle choices, education level, cognitive abilities, smoking, alcohol consumption, and existing comorbidities further influence the risk of overweight and obesity^[4]^. The World Obesity Federation issued a statement that obesity is a chronic relapsing disease^[6]^. It is not just a lifestyle problem and calls for the public to have an accurate understanding of obesity and destigmatize obesity.

## 1.2 Obesity-related comorbidities and their harms

Excessive weight and fat exert mechanical compression and mass effects to various organs, and the chronic inflammatory status caused by fat deposition also results in a series of metabolic damages. These changes can also affect body image and exercise ability, resulting in social discrimination (language, attitude and public speech) and a series of emotional and psychological issues, including low self-esteem. Obesity may lead to many chronic complications, including abnormal glucose metabolism (diabetes, prediabetes, and metabolic syndrome), dyslipidemia, hypertension and cardiovascular disease, chronic kidney disease, metabolic dysfunction-associated steatotic liver disease (MASLD), polycystic ovary syndrome (PCOS), female infertility, male hypogonadotropinism, obstructive sleep apnea syndrome (OSAS), asthma or reactive airway disease, osteoarthritis, tension urinary incontinence, gastroesophageal reflux disease, depression, anxiety and other mental and psychological disorders^[7]^. The risk of gout and cancer also increases with obesity^[8]^. In China, 70.7% of overweight patients and 89.1% of obese patients had at least one obesity-related complication^[9]^. The risk of developing one, two, or multiple complex obesity-related diseases in obese patients was 2.83 times, 5.17 times, and 12.39 times higher than that of normal weight, respectively. The degree of obesity is directly corrected with the risk of multiple comorbidities^[8]^.

Overweight and obesity are strongly associated with major chronic noncommunicable diseases^[10]^ and were identified as the sixth leading risk factor for death and disability in China by 2019^[3]^. For individuals over 40 who do not smoke, obesity may reduce life expectancy by an average of 5.8 years for men and 7.1 years for women^[11]^. In China, the medical expenses caused by overweight, obesity and its comorbidities have increased by about 21 times from 2000 (2.57 billion yuan) to 2009 (54.98 billion yuan)^[12-13]^. It is estimated that these costs could reach 418 billion yuan by 2030, accounting for about 22% of the country's total medical expenses^[14]^. Therefore, the rapid growth of obesity has brought great challenges to public health and clinical healthcare system in China.

## 1.3 Definition and diagnosis of obesity

Currently, the diagnosis of obesity remains controversial (Table 1). While clinical diagnosis typically relies on BMI, calculated from height and weight, this metric does not adequately reflect body composition or fat distribution. Comprehensive assessment requires additional measurements, such as waist circumference, hip circumference, body fat percentage, and visceral fat.

**Table 1** Commonly used diagnostic criteria for obesity in the Chinese population.

| Indexes | Test methods | Gender | Diagnostic criteria | | Merits and drawbacks |
| --- | --- | --- | --- | --- | --- |
| BMI | Body weight (kg)/height (m^2^) |  | Normal 18.5-23.9 kg/m^2^ | Overweight 24.0-27.9 kg/m^2^  Obesity≥28 kg/m^2^ | Simple operation, widely applicable, currently the most used diagnostic index; it does not reflect body composition or fat distribution. |
| Waist circumference |  | Male | Normal <85 cm | Abdominal obesity ≥90 cm | Partially reflect fat distribution and measure central obesity, simple operation and wide applicability; has large measurement error and requires correct measurement techniques. |
|  |  | Female | Normal <80 cm | Abdominal obesity ≥85 cm |  |
| WHR | Waist circumference/hip circumference | Male | Normal 0.85-0.90 | Abdominal obesity ≥ 0.90 |  |
|  |  | Female | Normal 0.75-0.80 | Abdominal obesity ≥ 0.85 |  |
| Waist height ratio | Waist circumference /height |  | Normal <0.5 | Abdominal obesity ≥ 0.5 |  |
| Body fat percentage | Bioelectrical impedance, dual-energy X-ray | Male | Normal 10%-20% | Obesity ≥25% | More accurate measurement, CT assessment of visceral fat area is considered the gold standard for diagnosing abdominal obesity; requires special equipment and is more complex and costly. |
|  |  | Female | Normal 15%-25% | Obesity ≥30% |  |
| Visceral fat area | CT, MRI |  | Normal＜80 cm^2^ | Abdominal obesity ≥80 cm^2^ |  |

BMI: body mass index; WHR: Waist circumference to hip circumference ratio. It is recommended that all medical institutions engaged in the diagnosis and treatment obesity adequately equip themselves with scales, measuring tapes, and body fat analyzers to assess BMI, waist circumference, and body fat percentage or visceral fat for all overweight and obese patients, including those with abdominal obesity.

## 1.4 Classification and staging of obesity

Obesity can be classified and staged using various methods, and there is currently no unified system. It can be categorized into simple obesity and secondary obesity based on etiology, and further divided into abdominal obesity and systemic obesity based on fat distribution. Abdominal obesity is particularly concerning, as it typically involves a significant increase in visceral fat, which is closely linked to a higher risk of obesity-related complications^[15]^. The American Association of Clinical Endocrinologists/American Endocrine Society (AACE/ACE) proposed adiposity-based chronic disease (ABCD) classification in 2017^[16]^. This system assesses the impact or potential health risks of obesity through an evaluation of underlying causes and associated complications, subsequently guiding treatment strategies based on these risks. This classification takes multiple factors such as etiology, degree of obesity, and complications into account. However, its complexity poses challenges for clinical application. Notably, while BMI remains a primary diagnostic criterion within this system, individuals with normal BMI but abnormal fat distribution (known as normal weight metabolic obesity) and their associated complications are often overlooked

Moreover, with the application of artificial intelligence (AI), scholars at home and abroad are trying to classify obesity with the assistance of AI. The Mayo Clinic uses machine learning methods to categorize obese patients into four phenotypes: "hungry brain", "hungry gut", "emotional hunger" and "slow burning", which are intended to inform treatment strategies. However, the necessary diagnostic indicators for these classifications can be overly complex, limiting their clinical applicability ^[17]^. A multi-center study in China proposed a new metabolic classification of obesity using AI and machine learning. This classification includes four subtypes: metabolically healthy obesity (MHO), hypermetabolic obesity-hyperuricemia (HMO-U), hypermetabolic obesity-hyperinsulinemia (HMO-I) and hypometabolic obesity (LMO). Each subtype exhibits distinct clinical characteristics and risk profiles for complications, showing good reproducibility and stability. Importantly, the indicators used for this classification are commonly applied in clinical practice, enhancing its reliability^[18-19]^. Appropriate obesity typing may aid in establishing treatment goals and selecting appropriate regimens. Clinicians can utilize different classifications to tailor approaches for obese patients based on individual needs, acknowledging the current lack of comprehensive evidence-based guidelines.

# 2. General principles for chronic management and diagnosis and treatment pathways for obesity

## 2.1 Principles of chronic obesity management

Chronic weight management aims to reduce the risk of obesity associated complications, controll the progression of existing complications, and improve overall quality of life and health. The primary goal of chronic management is to achieve and maintain an individualized optimal body weight over time (weight management encompasses various dimensions beyond just BMI) to improve long-term outcomes, such as cardiovascular health and overall mortality. Given the chronic and relapsing characteristics of obesity, management should follow the principles of timeliness, longevity, and individualization. The intervention should be initiated when a patient is overweight and/or experiences ongoing weight gain. An individualized treatment plan should be formulated according to the general condition, acceptance and compliance with weight management methods, necessitating long-term or even lifelong weight management. Chronic weight management typically includes an intensive treatment phase and a maintenance treatment phase. Different treatment goals and strategies should be formulated for the same individual to achieve reasonable long-term weight stability, prevent or reduce complications, and ultimately improve quality of life and prolong survival. It is crucial to recognize that even modest weight loss can confer significant health benefits, even if the ultimate treatment outcomes are not maintained long-term^[20-22]^.

## 2.2 Patient diagnosis and treatment pathways

The diagnosis and treatment pathways for obese patients are showed in Figure 1.


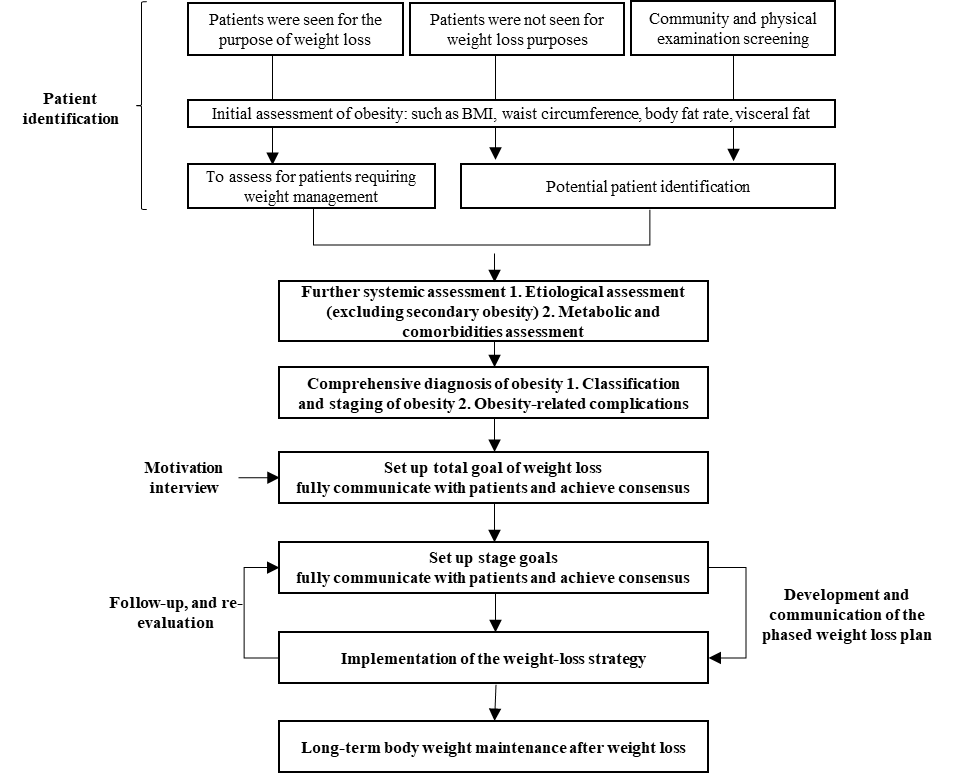


**Figure 1** Diagnosis and treatment pathway for obese patients.

### 2.2.1 Patient identification and initial assessment

Obese individuals often visit clinics for complications related to obesity or other health concerns. Clinicians should proactively identify potential obesity cases, especially among patients seeking care in various departments due to obesity-related issues. It's essential to assess whether these patients require referral to obesity specialists. Additionally, many individuals may avoid seeking medical treatment due to a lack of public awareness about obesity as a disease. Strengthening community screening and promoting regular physical examinations can help guide patients to seek appropriate care.

Initial identification of obesity includes visual assessment (i.e., the doctor's perception of the patient) and the measurement of anthropometric indicators (e.g., BMI, waist circumference, body fat percentage, visceral fat, etc., refer to Section 3 of Part 1 "Definition and Diagnosis of Obesity"). For abdominal patients with normal BMI, the assessment of body fat percentage and visceral fat is particularly important. A comprehensive evaluation is recommended for the diagnosis of obesity.

### 2.2.2 Whole-body assessment

#### (1) Etiological assessment

Clinicians should conduct a comprehensive assessment of the weight trajectory from birth, examining factors such as the timing and rate of significant weight gain, lifestyle changes (diet and exercise), family history of obesity, complications, comorbidities, and medication use. Identifying potential secondary factors of obesity is essential. Secondary obesity is characterized by excessive fat deposition resulting from underlying medical conditions or medications, distinct from simple obesity. Treatment for secondary obesity typically requires targeting the specific underlying condition, while conventional weight-loss approaches (e.g., lifestyle changes) may be less effective^[23]^. For suspected cases, especially those with a BMI of ≥40 kg/m^2^ and rapid increase in body weight, timely medical history review, specialist physical examination and auxiliary examinations for screening secondary obesity are needed. Referrals are referred when necessary. Genetic factors, neuroendocrine factors, and iatrogenic factors that lead to weight gain are common causes of obesity. Exogenous events experienced in an upbringing environment can also lead to significant changes in body weight in individuals. Common weight-influencing events in adulthood include pregnancy, schooling, marriage or divorce, decreased physical activity, eating disorders, and psychological problems^[24]^. Clinicians need to identify the impact of possible life events on the weight, and provide targeted counseling in the follow-up weight loss plan in the process of doctor-patient communication.

#### (2) Metabolic and comorbidity assessment

The assessment of obesity comorbidities is helpful for timely intervention. Some comorbid conditions may be subtle and easily overlooked, or patients may not recognize their relationship to obesity and may not express concerns. Given that obesity is a systemic disease, a comprehensive evaluation of comorbidities is essential. Comprehensive evaluation includes endocrine and metabolic system (e.g., diabetes, dyslipidemia), respiratory system (e.g., sleep apnea), cardiovascular system (e.g., coronary heart disease, hypertension), urinary system (e.g., chronic kidney disease, tension incontinence), reproductive system (e.g., polycystic ovary syndrome, infertility), digestive system (e.g., gastroesophageal reflux disease, MASLD), skeletal motor system (e.g., osteoarthritis), psychological disorders (e.g., depression, anxiety) and tumors aspects. Evaluations typically begin with open-ended questions to uncover the patient's health concerns, which often reflect the most distressing comorbidities. These concerns can serve as significant motivators for obesity treatment. Clinicians should conduct a comprehensive inquiry based on the patient's responses and perform additional measurements to confirm diagnoses of comorbidities.

### 2.2.3 Comprehensive diagnosis of obesity

A comprehensive diagnosis of obesity is established based on the results from the assessments above. This includes the classification and staging of obesity and the identification of obesity-related comorbidities (see Chapter 1, Section (3) " Definition and diagnosis of Obesity" and Section (4) "Classification and Staging of Obesity").

### 2.2.4 Motivational interviewing

Motivational interviewing is a key component of the communication between clinicians and patients to set up shared goals. This approach promotes patients' intrinsic motivation and improves patients' compliance, thereby improving treatment effectiveness, quality of life and satisfaction. During motivational interviews, clinicians identify the most burden of obesity on the patient's life and collaboratively set individualized weight loss goal. Clinicians need to express empathy, highlight differences, encourage patients’ expression, and uncover critical challenges faced during weight loss. Together with the patient, they should select manageable targets for treatment. It is vital for clinicians to bolster patients' intrinsic motivation, avoid confrontations, address resistance, and assess confidence in treatment. Support from family and friends can also play a pivotal role in helping patients develop a reasonable, patient-centered weight loss plan.

### 2.2.5 Weight-loss goals

The long-term goal of obesity treatment is to achieve and maintain an individualized optimal weight, enhancing long-term outcomes (e.g., cardiovascular outcomes, all-cause mortality) and improve overall quality of life. The individualized optimal weight is defined as the weight that maximizes long-term physical and mental health, generally involving a multidimensional normalization of weight (including BMI, waist circumference, body fat percentage, etc.)^[25]^. Appropriate adjustments can be made according to the individual characteristics (e.g., age, complications) in clinical practice. The realization of long-term goals is typically divided into two stages: intensive treatment phase and maintenance treatment phase (Fig. 2).

**(1) Intensive treatment period:**

The intensive treatment period spans from initiation treatment to the point closest to individualized optimal body weight. Considering most obesity has a long treatment cycle and a long time to achieve individualized optimal weight, it is essential to timely adjust the treatment plan to enhance behavioral incentives, improve compliance and control reasonable weight change trajectory. This period can be decomposed into several stages according to individual conditions with multiple short-term goals established to facilitate gradual progress. Milestones may include achieving specific changes in obesity assessment indicators (e.g., weight, waist circumference, body composition) or controlling and improving complications. Individualized disease burden identified in motivational interviews can also be used as milestones for weight management. Each stage can range from a few weeks to a few months, but typically should not exceed three months. For example, the first stage goal may aim for 10%-15% weight loss within 3-6 months for patients who are young and have few complications. It helps build patient confidence and improve adherence to subsequent bariatric treatments. Conversely, for older patients or those with significant complications, a slower stage goal, such as losing 5%-10% of weight over 3 to 6 months, may be more appropriate to ensure safety and minimize side effects. The next stage goal is set to gradually achieve an individualized optimal weight after achieving the first stage goal.

**(2)** **Maintenance treatment period:**

The maintenance treatment phase refers to the long-term maintenance phase after the optimal weight that is closest to individualization. The primary goal of this phase is to maintain the weight at the individualized optimal level over long term, minimize weight fluctuations and achieve long-term weight management goals.


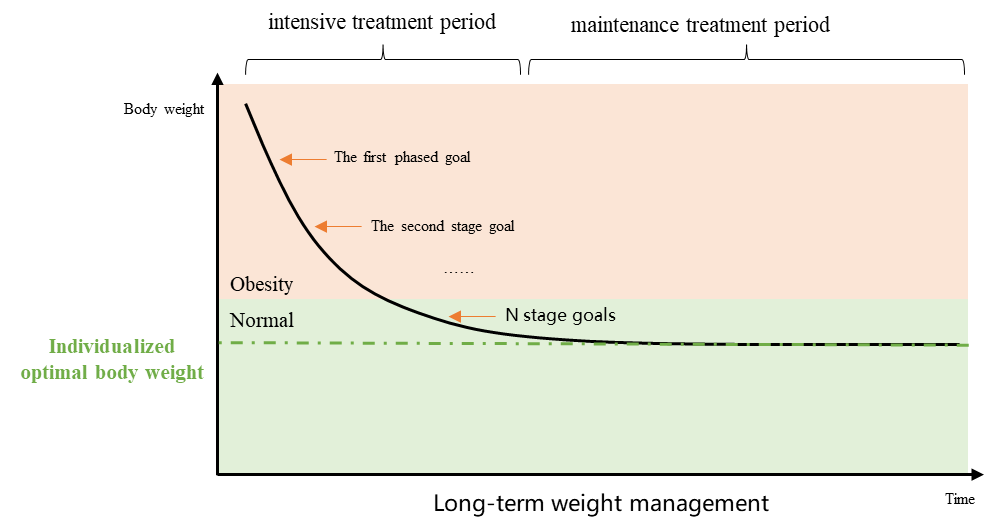


**Figure 2** Stages of long-term weight management

Individualized optimal body weight refers to the weight that makes long-term physical and mental health optimal, and for most patients, it is a multidimensional weight normalization (including BMI, waist circumference, body fat percentage, etc.). In clinical practice, appropriate adjustments can be made according to the individual characteristics (e.g., age, complications, etc.). The first phase goal can be to lose 10% to 15% of body weight within 3 to 6 months for patients who are young, have few complications, or are at risk for complications only. The first stage goal can be set to lose 5%-10% of weight from 3 to 6 months and lose weight steadily for older patients with more complications.

## 2.3 The overall principles of the implementation of weight-loss strategies and long-term maintenance

The earlier weight management interventions are initiated, the greater the potential benefits^[26-27]^. Therefore, weight management should be proactive, starting as soon as weight gain is detected rather than waiting for complications to develop or worsen. While lifestyle interventions are essential throughout long-term weight management, they are often insufficient on their own or may lead to weight regain^[28]^. For patients with obesity or abdominal obesity, or those with obesity-related comorbidities, it is recommended to initiate pharmacotherapy during the intensive treatment phase. Medications should be considered promptly if lifestyle interventions fail to produce satisfactory results (e.g., less than 5% weight loss within three months) in patients who remain overweight without comorbidities.

The implementation of the weight-loss strategy requires fully communication with the patient to understand their needs and concerns. This approach helps to formulate the most suitable plan that the patient is more likely to follow effectively. It is essential to guide the patient through the weight-loss process, including self-monitoring and follow-up plans, to enhance the effectiveness of the strategy. A phased follow-up plan should be developed to evaluate treatment efficacy, monitor adverse reactions, and make timely adjustments to the treatment regimen. This will improve patient compliance and support the achievement of weight-loss goals. Maintaining weight loss can be challenging after the intensive treatment phase, necessitating ongoing comprehensive weight management therapy. Depending on the patient's compliance and the effectiveness of weight maintenance, lifestyle interventions alone or in combination with weight-loss medications may be appropriate. For specific methods, please refer to "Methods for long-term weight management" (Table 3). For patients who are not suitable for conservative treatment and meet the indications for surgery, the option of metabolic surgery can be discussed with a bariatric specialist if necessary (Fig. 3).


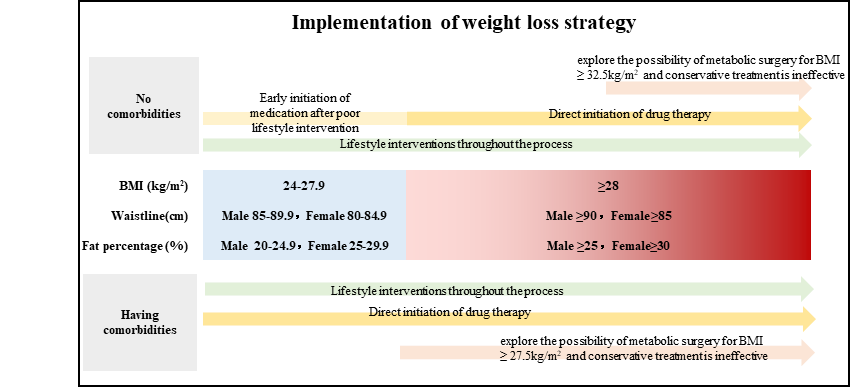


**Figure 3** General principles for the implementation of weight-loss strategies.

# 3. Lifestyle interventions in long-term weight management

Lifestyle interventions are the cornerstone of long-term weight management, whether during intensive or maintenance treatment. These interventions encompass dietary management, exercise interventions, habit formation, and psychosocial support. It is worth noting that lifestyle interventions for obesity are highly individualized, and patient compliance significantly influences their effectiveness^[28-29]^. Therefore, it is recommended that multidisciplinary clinicians engage in discussions with patients to create personalized intervention plans that are easy for them to accept and adhere to. Adjustments should be made based on patient’s existing lifestyle preferences and habits, as this is a key principle of effective lifestyle intervention.

## 3.1 Diet management

The significance of diet management extends beyond reducing energy intake to effectively promote weight loss; it also plays a crucial role in improving metabolic disorders such as blood glucose, blood pressure, lipids profiles, and insulin resistance. Achieving clinically meaningful weight loss hinges on limiting total caloric intake and maintaining a negative balance between energy intake and expenditure. Additionally, diet composition, eating methods, and timing are important factors in weight loss process^[30]^. There are many forms of dietary patterns, such as energy-restricted diets, low-carbohydrate diets, high-protein diets, intermittent diets, and meal replacements (Table 2). The effectiveness of these approaches varies greatly among individuals, with short-term weight loss ranging from 1% to 16.1%. However, maintaining an individualized optimal body weight for a long time using a single dietary management can be challenging, and most patients will rebound within 6 to 12 months of intervention^[41,45-46]^.

**Table 2** Summary of common dietary strategies for weight management.

| Diet category | Implementation | Effect and benefit |
| --- | --- | --- |
| Energy-restricted diet | An energy-limited diet is to limit daily energy intake with less than the energy required, usually limiting 1200-1500 kcal/d for women, 1500-1800 kcal/d for men, or 500 or 750 kcal/d or 30% less energy intake based on estimated personal energy needs. The more stringent very-low-energy diet is the special type of energy-limited diet, which controls the energy intake level within 800 kcal/d ^[31].^ The energy supply ratio of macronutrients is consistent with a balanced dietary pattern (40%-55% carbohydrate, 15%-20% protein, 20%-30% fat)^[31].^ | It can effectively reduce body weight and fat content, improve insulin resistance and other components of metabolic syndrome, and reduce the risk of cardiovascular disease^[32-34].^ |
| Low-carbohydrate diet | Low-carbohydrate diet usually refers to the dietary pattern ^[31,35-36]^ in which the daily carbohydrate energy supply ratio is 20%–40% of the total daily energy per day. Very low carbohydrate diet (also known as ketogenic diet) is a special type of low carbohydrate diet, which means controlling the carbohydrate energy ratio within 20%^[31,36]^. | The application of low carbohydrate diet in the short term can significantly reduce bodyweight, and can effectively improve blood glucose, lipids and other indicators ^[37-38]^. However, the low-carbohydrate diet has a low compliance profile, and fewer studies have evaluated its long-term weight loss effects and adverse outcomes. |
| High-protein diet | A high-protein diet usually refers to a daily protein energy supply ratio of more than 20% of the total daily energy, but generally not more than 30% of the total daily energy of the dietary pattern^[31,36]^. | It helps to reduce body weight, improve blood glucose and lipid and other cardiovascular disease risk factors. Some research evidence shows that a high-protein diet attenuates intestinal lipid absorption and prevents fat increasing^[39]^. It is an effective strategy to prevent the rebound^[40]^. |
| Intermittent diet | (1) alternate day diet: including normal eating days and diet days alternate. On feeding days, patients can eat freely, with no limit on the type or quantity of food; on diet days, patients only eat 0-25% (500-800 kcal), a diet day meal can be eaten at one time or scattered throughout the day, without affecting the weight loss effect; (2) 5:2 intermittent diet: it is a modified version of alternate day diet, with 5 normal eating days and 2 diets per week, which can be continuous or discontinuous; (3) time-limited dieting: limit eating to a specific time (usually 4-12 hours) without limiting energy intake; drink only zero-calorie drinks in the non-eating time. | Achieve light to moderate weight loss (3%-8% decrease from baseline) over a short period^[41]^ (8-12 weeks). Some studies suggest that intermittent diet may improve cardiometabolic risk factors such as blood pressure, blood lipids, insulin resistance and HbA1c. |
| Mediterranean diet | There is no uniform standard for the mediterranean diet pattern, which is characterized by higher intake of olive oil, nuts, whole grains, fruits and vegetables, moderate consumption of red wine, and reduced intake of red meat or processed food^[42]^. | It can effectively reduce the body weight and waist circumference^[43]^. |
| Dietary approaches to stop hypertension（DASH） | DASH emphasizes increasing the intake of more vegetables, fruits, low-fat (or skimmed) milk, taking whole grains, reducing the intake of red meat, fat, refined sugar and sugar-sweetened drinks, and eating a moderate number of nuts. This diet provides rich minerals such as potassium, magnesium and calcium, as well as dietary fiber, increasing the intake of high-quality protein and unsaturated fatty acids. | It can effectively reduce the body weight, blood pressure, blood glucose and blood lipid，and improve insulin resistance^[44]^. |

## 3.2 Exercise intervention

Exercise interventions are crucial components of comprehensive lifestyle modifications for long-term management. These include supervised and unsupervised exercise, occupational activities, housework, personal care, commuting, and leisure activities. Whereas exercise interventions include aerobic exercise, resistance exercise, aerobic combined resistance exercise, etc. While exercise alone may result in modest weight loss (about 2.4%), it is essential for long-term weight maintenance and improving quality of life. Additionally, it helps reduce obesity-related metabolic disorders and cardiovascular complications^[47-48]^. However, precautions are necessary to protect joints and prevent sports-related injuries, especially since obesity-related conditions like arthritis can hinder exercise and impact its effectiveness.

## 3.3 Behavior modification

Behavior management is a vital part of any weight-loss plan and is essential for success, though it can be challenging to adhere to. Professionals should guide patients in setting specific targets for calorie intake, time length of physical activity, and frequency of self-monitoring. This helps obese patients make objective, measurable changes in their diet, activity, and related behaviors^[49-50]^. Self-monitoring allows patients determine their own dietary patterns and establish goals for reducing calorie intake^[51]^. Regular visits, individualized treatment, and long-term maintenance are crucial during habit formation. Structured behavioral therapy programs have been shown to reduce body weight by an average of 7 kg-10 kg in the first six months. However, the efficacy varies widely among individuals, often requiring high-intensity intervention^[52-53]^.

## 3.4 Psychosocial support

Hedonic eating and eating disorders contribute to obesity, which usually requiring drugs or cognitive-behavioral therapy. Certain psychotropic drugs (e.g., paroxetine, quetiapine) may increase weight by increasing appetite^[54-55]^. Additionally, patients with obesity often experience negative emotions, including low self-esteem and self-blame, which can lead to depression or bipolar disorder, further aggravating eating disorders. Thus, psychological assessment is an essential aspect of obesity treatment and can be combined with medication, psychology, and cognitive behavioral therapy if necessary^[56]^. Studies indicate that family-based behavioral weight management can help patients develop good dietary, exercise, and lifestyle habits, potentially leading to 5%-20% weight loss^[57]^. In clinical practice, social support is crucial for obese patients to adhere to healthy behaviors over the long term, incorporating support from family, friends, healthcare providers, and social groups, such as support networks for individuals with obesity.

# 4. Weight-loss drug therapy in long-term weight management

With the progress in drug development, pharmacotherapy has emerged as a crucial option for long-term weight management. The efficacy and safety of new drugs, along with their metabolic and cardiorenal benefits beyond weight loss, have brought new breakthroughs to clinical practice.

For most patients who are overweight or obese (including abdominal obesity), medications can be initiated directly when indicated. This is particularly true for those who have struggled to lose weight or maintain weight-loss. In cases where lifestyle interventions prove ineffective (e.g., less than 5% weight loss over three months), drug therapy may also be considered for patients with mild obesity (BMI 24 to 27.9 kg/m²) and no significant comorbidities. An individualized, comprehensive assessment of each patient in clinical practice is essential, especially for those at high risk for adverse drug effects or with poor tolerance (such as the elderly taking multiple drugs, or those with severe liver and kidney dysfunction). It is necessary to utilize weight-loss drugs judiciously under the guidance of doctors after fully evaluation.

Doctors need to choose drugs that are suitable for patients, ensuring high acceptance and adherence. Fully communication about the medication methods, potential side effects, and follow-up plans is vital during drug treatment. To prevent treatment inertia, regular monitoring of body weight and metabolic indexes regularly is necessary, along with timely evaluations of efficacy and adjustments in drug dosages. Effective weight loss not only boosts confidence and improve adherence but also ensures better outcomes for patients. Lifestyle interventions remain the cornerstone of long-term weight management which need to be combined with the usage of medications. Combining these strategies can help individuals adhere to lifestyle changes more effectively and facilitate positive behavioral modifications.

## 4.1 Overview of the development of weight-loss drugs

The development of anti-obesity drugs (AOMs) began in the 1990s, and after over a century of development, most of these drugs have been withdrawn from the market due to safety problems. Notable examples include desiccated thyroid extract (DTE) (increasing the risk of arrhythmia and cardiac arrest), 2, 4-dinitrophenol (causing agranulocytosis and fatal hyperthermia), sympathomimetic drug amphetamine (known for its addictive properties), and "rainbow pills" which was the combined amphetamine, methamphetamine and amobarbital (leading to cardiotoxicity or sudden death). Additional drugs that have faced similar issues are amphetamine analogues-aminorex (associated with pulmonary hypertension), appetite suppressant phenylpropanolamine (linked to intracranial hemorrhage and stroke), fenfluramine (causing pulmonary hypertension and valvular disease), the serotonin and norepinephrine reuptake inhibitor(SNIR) sibutramine (which poses cardiovascular disease risks), the cannabinoid receptor antagonist rimonabant (connected to depression and suicide), and serotonin 2C receptor agonist lorcaserin (associated with increased cancer risk)^[58-59]^. Today, only a few traditional weight-loss drugs are still used globally, such as phentermine, amfebutamone, orlistat, and some combination formulations like phentermine/topiramate and naltrexone/amfebutamone. In China, orlistat is the only approved traditional weight-loss drug for weight management.

In addition to safety considerations, effectiveness was also a key factor. In 2007, the U.S. Food and Drug Administration (FDA) established guidelines for the development of weight-loss drugs, setting criteria for efficacy. Specifically, a drug must demonstrate a net weight loss of at least 5% compared to placebo after one year of treatment, with the proportion of subjects losing ≥5% body weight in the drug group exceeding that of the placebo group by more than double^[60]^. This led to a new era of stricter regulation in the research and development of weight-loss medications.

New drugs targeting nutrient-stimulated hormone (NuSH) receptor have brought hope to the exploration of weight-loss drugs in recent years^[61]^. NuSH refers to hormones secreted in response to dietary nutrients that regulate appetite and metabolism, including glucagon-like peptide-1 (GLP-1) and glucose-dependent insulinotropic peptide (GIP). These new weight-loss drugs include: (1) Single-receptor agonists, mainly GLP-1 receptor agonists (GLP-1RAs), the daily preparation liraglutide was approved by the FDA for weight management in 2014, the one weekly preparation semaglutide was approved by the FDA for weight management in 2021. Both liraglutide and benaglutide are also approved for weight management in China. (2) dual-receptors agonists, such as the new drug GIP/GLP-1 dual receptor agonist tirzepatide, which has shown over 20% weight reduction, offering greater weight loss potential than single-receptor agonists. The FDA approved it for obesity in 2023, and it is approved for obesity in China in 2024. In addition, GLP-1/GCG (glucagon) dual receptor agonists and GLP-1/amylin agonist combinations are currently under active development in phase 3 clinical trials. (3) Three-receptor agonists, GLP-1/GIP/GCG three-receptor agonists are still in phase 3 clinical trials, and may provide even greater weight loss potential.

## 4.2 Introduction to the classification of weight-loss drugs

### 4.2.1 Lipase inhibitors

The only lipase inhibitor available on the market is orlistat at present. This medication inactivates lipase by targeting the active sites of gastric and pancreatic lipase, thereby inhibiting the hydrolysis of triglycerides into absorbable free fatty acids and monoacylglycerol. This mechanism affects the absorption of triglycerides and ultimately reduces caloric intake^[62-63]^.

Orlistat was approved by the U.S. FDA in 1999 and received approved for obesity management in China in 2001. It is the only over-the-counter weight loss drug approved in China, with a recommended dose of 120 mg taken three times a day, either with or within one hour after meals^[64]^. Pharmacotherapy with orlistat is recommended in conjunction with lifestyle and behavioral interventions.

Orlistat is associated with mild weight loss, resulting in an additional 3.16% weight loss compared to lifestyle interventions alone in patients with overweight or obesity^[65]^. Over one-year period, weight loss typically ranges from 2.9 to 4.4 kg, with most loss occurring in the first six months of treatment. Weight loss is dose-dependent, although there is no evidence that doses greater than 120 mg three times a day yield better results^[66]^. Additionally, orlistat may reduce the incidence of obesity-related risk factors and diseases, including type 2 diabetes, insulin resistance, hypercholesterolemia, hypertension, and nonalcoholic fatty liver disease, while also promoting a reduction in fat content. Its effectiveness can be sustained with long-term use^[67-68]^.

The primary adverse reactions associated with orlistat are gastrointestinal, stemming from its pharmacological effects that prevent fat absorption. Common side effects include stool urgency, fatty stools, steatorrhea, increased stool frequency, fecal incontinence, oily spots, increased gas, and abdominal pain/abdominal discomfort. There is also a potential risk of malabsorption of fat-soluble vitamins (A, D, E, K) and certain drugs (e.g., cyclosporine, thyroid hormones, anticonvulsants). It is important to consider patient's personal values and preferences when prescribing orlistat, and to thoroughly explain potential adverse reactions and coping strategies. Patient education should include dietary guidelines to minimize fat-rich food and regular multivitamin supplementation. Care should be taken to assess any concurrent medications to avoid interference with the treatment of other conditions.

### 4.2.2 NuSH receptor agonists

#### (1) NuSH single receptor agonists

The only NuSH single receptor agonists on the market in China are GLP-1RAs and their analogues. GLP-1 as an incretin hormone is stimulated by nutrients (such as carbohydrates, lipids, etc.) in the gastrointestinal tract and is secreted by L cells in the small intestine. GLP-1 receptors are widely distributed in the central and peripheral areas, including the hypothalamus, gastrointestinal tract, and pancreatic islet. Activation of GLP-1R regulates hypothalamic appetite, influences the reward system to control eating behavior, and delays gastric emptying, thereby exerting weight loss^[69-72]^. GLP-1RA mimics the effects of natural GLP-1 to reduce weight.

The GLP-1RAs approved for weight management in China mainly include short-acting benaglutide (approved in 2023, administered multiple times daily), daily formulation liraglutide (approved in 2023), and once weekly formulation semaglutide (approved in 2024), all of which are administered via subcutaneous injection. Oral GLP-1RA preparations (such as oral semaglutide and Orforglipron) are currently in Phase 3 clinical development for weight management indications in China. Other GLP-1RAs, like dulaglutide, exenatide, lisinatide and losenatide, lack indications for weight management or have not conducted clinical trials (only for type 2 diabetes mellitus), thus are not recommended for long-term weight management in overweight or obese patients.

1. **Benaglutide (injectable):** This short-acting GLP-1RA is titrated for weight-loss therapy at 0.06 mg three times daily in week 1, the dose is increased to 0.10 mg each time in week 2, increased to 0.14 mg each time in week 3, and increased to 0.20 mg each time in week 4 and maintained for weight loss therapy. The recommended maintenance dose is 0.20 mg three times daily or the maximum tolerated dose. In phase 3 clinical trial involving overweight/obese (non-diabetic) individuals, benaglutide (0.2 mg three times daily for 16 weeks) resulted in an average weight loss of 6.0% (significantly higher than the 2.4% observed in the placebo group)^[73]^.
2. **Liraglutide (injectable):** This daily formulation starts at 0.6 mg subcutaneously, with a daily dose of 0.6 mg incremented at weekly intervals, and a daily dose of 3 mg or the maximum tolerated dose is recommended and maintained. In the global Phase 3 SCALE study enrolled overweight/obesity(non-diabetic) subjects, participants receiving liraglutide 3.0 mg once daily experienced an average weight loss of 8.0% over 56 weeks, compared to 2.6% in the placebo group. For overweight/obese individuals with T2DM, liraglutide dose of 3.0 mg and 1.8 mg once-daily treatment for 56 weeks had mean weight loss of 6.0% and 4.7%, respectively (significantly higher than 2.0% in the control group)^[75]^. The results of phase 3 clinical trials of liraglutide in overweight/obese Chinese population have not been disclosed at present.
3. **Semaglutide (injectable):** The once weekly formulation of GLP-1RA for weight loss therapy is used as a starting dose of 0.25 mg subcutaneously once weekly for the first four weeks, increased to 0.5 mg once weekly for the second four weeks, 1.0 mg for the third four weeks, 1.7 mg for the fourth weeks, and 2.4 mg after week 17 and maintained for weight loss. The recommended maintenance dose is 2.4 mg once weekly or the maximum tolerated dose. In the global Phase 3 STEP series, semaglutide 2.4 mg once weekly subcutaneously reduced an average body weight of 16.9% after 68 weeks treatment in overweight/obese people (non-diabetic) (compared to 2.4% in the placebo group)^[76]^. For overweight/obese T2DM patients, semaglutide 2.4 mg once weekly subcutaneously reduced an average body weight of 10.6% after 68 weeks (compared to 3.1% in the placebo group)^[77]^. In the phase 3 clinical trial in an overweight/obese Chinese population (with or without T2DM), participants lost an average of 12.8% of their body weight after 44 weeks (compared to 3.0% in the placebo group)^[78]^.
4. **Semaglutide tablets (oral formulation):** This oral preparation uses sodium caprylate to promote semaglutide absorption into the bloodstream through the gastric mucosa^[79]^. Due to low oral bioavailability and the absorption/pharmacokinetic variability is extremely high, there are strict requirements at the time of administration, requiring the drug to be taken on an empty stomach and at least half an hour after taking the drug before eating, drinking, or other medications. The initial weight-loss dose is 3 mg once daily for the first four weeks, 7 mg daily for the second weeks, 14 mg for the third weeks, 25 mg for the fourth weeks, and 50 mg after week 17 and maintained for weight loss. In the OASIS series of a global Phase 3 clinical trial, the average weight loss of subjects in overweight/obese people (non-diabetic) was 15.1% after 68 weeks of treatment with oral semaglutide 50 mg once weekly (compared to 2.4% in the placebo group)^[80]^. Currently, phase 3 clinical trials of oral semaglutide in overweight/obese Chinese populations are still ongoing.
5. **Orforglipron (Oral Formulation):** This small molecule non-peptide GLP-1RA can been taken with food and liquids without affecting absorption^[81]^. In global phase 2 clinical trial, orforglipron showed average weight losses of 8.6%-12.6% (2.0% in the placebo group) and 9.4%-14.7% (2.3% in the placebo group) in overweight/obese people (non-diabetic) treated with orforglipron 12, 24, 36 or 45 mg once daily for 26 weeks^[82-83]^. It is currently in phase 3 clinical trial.

#### (2) NuSH dual-receptor agonists

The gradual understanding of NuSH and the successful application of its single receptor agonist in weight management have promoted the further development of weight-loss drugs based on NuSH multi-receptor modulation, to achieve better weight loss outcomes with long-term medication. The GIP/GLP-1 dual receptor agonist tirzepatide is currently the only approved agent for obesity management s in Europe，the United States and China. The GLP-1/GCG dual-receptor agonists mazdutide and survodutide, and the GLP-1/amylin receptor agonist combination cagrisema are still undergoing phase 3 trials.

###### GIP/GLP-1 dual receptor agonists

Both GIP and GLP-1 are incretin hormones stimulated by nutrients and secreted by intestine in a physiological state. GIP receptor (GIPR) can not only produce some biological effects like GLP-1R activation (such as central appetite suppression, increase peripheral insulin sensitivity, etc.), but also act on adipose tissue to regulate lipid storage and fat mobilization. The combined agonism of GLP-1R and GIPR may have a unique synergistic effect on weight regulation through complex synergistic complementarity^[84]^.

Tirzepatide is the first and only GLP-1/GIP dual agonist, which has been approved for long-term weight management in Europe and the United States in 2023, and in China in 2004. Tirzepatide is a once weekly formulation of 2.5 mg subcutaneously once weekly starting at weeks 1 to 4, then increasing by 2.5 mg every 4 weeks, titrating to 15 mg or the maximum tolerated dose and maintaining it for a long time. The global phase 3 clinical trial SURMOUNT series showed that for overweight and obese patients (non-diabetic), the average weight loss of tirzepatide 5 mg, 10 mg, and 15 mg once weekly treatment for 72 weeks was 16.0%, 21.4% and 22.5% respectively (2.4% weight loss in placebo group)^[85]^. For patients with overweight/obesity and T2DM, the mean weight reduction of tirzepatide 10 mg and 15 mg once weekly after 72 weeks treatment was 12.8% and 14.7% respectively (compared to 3.2% in placebo group)^[86]^. In the Chinese population of overweight and obese patients (non-diabetic), the average weight loss of tirzepatide 10mg and 15mg once weekly for 52 weeks was 14.4% and 19.9%, respectively (compared to 2.4% in the placebo group)^[87]^. In addition, the results of indirect comparative studies showed that the GIPR/GLP-1R dual agonist (tirzepatide 15 mg) had a more significant weight loss than GLP-1R single agonist (semaglutide 2.4 mg) (mean weight loss difference of - 5.92%)^[88]^.

###### GIP/GCG dual receptor agonists

Glucagon (GCG) is a polypeptide hormone synthesized and secreted by pancreatic α cells, which can not only promote glycogenolysis and gluconeogenesis, but also promote lipolysis and fatty acid oxidation by activating lipase, resulting in a beneficial effect on energy expenditure^[89]^. GCG and GLP-1 can synergistically reduce food intake and increase energy expenditure, and GLP-1 can balance the increased blood glucose caused by GCG. The GLP-1/GCG dual receptor agonist that in phase 3 clinical trial is mazdutide. For overweight/obese Chinese population (GLORY-1), mazdutide 4, 6mg once weekly for 48 weeks reduced body weight of 12.05% and 14.84% (compared to 0.47% in the placebo group)^[90]^. Results from the global Phase 2 clinical study showed that the average weight loss of survodutide 3.6 mg and 4.8 mg once weekly for 46 weeks was 13.2% and 14.9%, respectively (compared to 2.8% in the placebo group)^[91]^.

###### GLP-1/amylin receptor agonist combination

Amylin is a type of peptide hormones secreted by β cells of pancreatic islet. The physiological functions include regulating energy intake and food preference, delaying gastric emptying, and synergistically regulating blood glucose homeostasis^[92]^. Cagrilintide is a long-acting amylin analogue that is expected to provide greater weight loss than semaglutide alone in combination with GLP-1RA semaglutide. Phase 3 clinical trials are currently ongoing^[93]^.

#### (3) NuSH three-receptor agonists

The GLP-1/GIP/GCG three-receptor agonists are the only NuSH three-receptor agonists under development. Retatrutide what is once weekly injection preparation is the only agent in phase 3 clinical trial. In the global phase 2 clinical trial, the average weight loss of retatrutide 4 mg, 8 mg, and 12 mg weekly for 24 weeks was 12.9%, 17.3%, and 17.5% (compared to 1.6% in the placebo group), and the average weight loss at 48 weeks was 17.1%, 22.8%, and 24.2%, respectively (compared to 2.1% in the placebo group) in overweight/obese people (non-diabetic)^[94]^. Phase 3 clinical trials are currently ongoing.

Existing and upcoming weight-loss drugs are listed in Table 3 for reference.

**Table 3** Existing and upcoming weight-loss drugs.

| Classification | Weight-loss drugs | Mechanism | Mode of administration and recommended dose | Weight loss effect | Safety | Marketing and indications |
| --- | --- | --- | --- | --- | --- | --- |
| Lipase inhibitors | Orlistat | Lipase inhibitors | 120 mg, orally, 3 times daily, at meals or within 1 h after a meal | Global (overweight/obesity): 3.16% additional weight loss, lost 2.9 to 4.4 kg one year, most of the weight loss occurred in the first 6 months of treatment^[65]^ | It mainly causes gastrointestinal adverse such as oily spot, increased gastrointestinal exhaust, urgency fecal sense, fat stool, fat diarrhea, increased stool frequency and fecal incontinence | It was marketed as an OTC drug in China in 2001. |
| NuSH single receptor agonists | Benaglutide | GLP-1RA | 0.2 mg or maximum tolerated dose, subcutaneous injection,  three times daily | Chinese population (overweight/obesity non-diabetic): weight loss of 6.0% with 0.2 mg three daily for 16 weeks^[73]^ | Gastrointestinal reactions including nausea, diarrhea, constipation, vomiting, indigestion, abdominal pain, decreased appetite, these adverse reactions are generally mild to moderate, common in the initial treatment and dose increasing period, gradually reduced with the extension of treatment.  Other adverse reactions: hypoglycemia risk increased combined with sulfonylurea or insulin, elevated amylase and lipase, cholelithiasis, increased heart rate, allergic reaction, acute pancreatitis, but the incidence of these reactions are low.  GLP-1RA may induce thyroid C cell tumors in rodents, although there is no sufficient clinical evidence that GLP-1RA causes human thyroid C cell tumors. With or with family history of medullary carcinoma of thyroid or multiple endocrine adenomatosis type 2 are contraindications for this class of drugs | Approved for T2DM in 2016 and approved for weight management in 2023 in China |
|  | Liraglutide | GLP-1RA | 3 mg or maximum tolerated dose, subcutaneous injection, once daily | Chinese population: no data; global (overweight/obese non-diabetic): weight loss of 8.0% with 3.0 mg daily for 56 weeks ^[74]^; global (overweight/obese T2DM): weight loss of 6.0% and 4.7% with 3.0 mg and 1.8 mg daily for 56 weeks mean, respectively^[75]^ |  | Approved for T2DM in 2011 and approved for weight management in 2023 in China |
|  | Semaglutide | GLP-1RA | 2.4 mg or maximum tolerated dose, subcutaneous injection, once weekly | Chinese population (overweight/obesity with or without T2DM): weight loss of 12.8% 2.4 mg once weekly for 44 weeks^[78]^; global (overweight/obese non-diabetic): weight loss of 16.9% with 2.4 mg once weekly for 68 weeks ^[76]^; global (overweight/obese T2 DM): weight loss of 10.6% with 2.4 mg once weekly for 68 weeks^[77]^ |  | Approved for T2DM in 2021 and approved for weight management in 2024 in China |
|  | Semaglutide tablets | GLP-1RA (Oral formulation) | 50 mg or maximum tolerated dose, orally, once daily; take the medicine on an empty stomach in the morning and eat, drink liquid, or taking other oral drugs after half an hour | Chinese population: no data; global (overweight/obesity non-diabetic): weight loss of 17.4% with 50 mg once weekly for 68 weeks^[80]^ |  | Approved for T2DM in 2024 and is still in the phase 3 clinical trials for weight management in China |
|  | Orforglipron | GLP-1RA (Oral formulation) | Orally, once daily | Chinese Population: no data; global phase 2 clinical trial data (overweight/obesity non-diabetic): average weight loss of 9.4% to 14.7% with 12,24,36 or 45 mg once daily for 36 weeks^[82- 83]^ |  | In the phase 3 clinical trials |
| NuSH dual-receptor agonists | Tirzepatide | GIP/GLP-1 dual receptor agonists | 15 mg or maximum tolerated dose, subcutaneous injection, once weekly | Chinese population (overweight/obese non-diabetic): weight loss of 14.4% and 19.9% with 10,15 mg for 52 weeks^[87]^; global (overweight/obese non-diabetic): weight loss of 16.0%, 21.4%, 22.5% with 5, 10,15 mg for 72 weeks^[85]^; global (overweight/obese T2DM): weight loss of 13.4% and 15.7% with 10,15 mg foe 72 weeks, respectively^[86]^ |  | Approved for T2DM in 2024 and is approved for weight management in 2004 in China |
|  | Mazdutide | GIP/GCG dual receptor agonists | Subcutaneous injection, once weekly | Chinese population (overweight/obesity non-diabetic): weight loss of 12.05% and 14.84% with 4,6 mg for 48 weeks^[90]^ |  | The weight management indication is still in the application |
|  | Survodutide | GIP/GCG dual receptor agonists | Subcutaneous injection, once weekly | Chinese population: no data; global phase 2 clinical trial (overweight/ obesity non-diabetes): weigh loss of 13.2% and 14.9% with 3.6 mg and 4.8 mg for 46 weeks, respectively^[91]^ |  | In the phase 3 clinical trials |
|  | CagriSema | GLP/Amylin receptor agonist combination | Subcutaneous injection, once weekly | No data |  | In the phase 3 clinical trials |
| NuSH three-receptor agonists | Retatrutide | GLP-1/GIP/GCG three-receptor agonists | Subcutaneous injection, once weekly | Chinese population: no data; global phase 2 clinical trial data (overweight/obesity non-diabetic): weight loss of 17.1%, 22.8% and 24.2% with 4,8 and 12 mg for 48 weeks, respectively^[94]^ |  | In the phase 3 clinical trials |

OTC: over-the-counter; NuSH: nutritional stimulating hormone; GLP-1: glucagon-like peptide-1; GCG: glucagon; GIP: glucose-dependent insulinotropic peptide

## 4.3 Extra-weight loss benefits of NuSH receptor agonists

NuSH receptor agonists enhance multi-dimensional body mass index through both direct or indirect effects while promoting weight loss, leading to multiple benefits such as improved metabolism and reduced comorbidities.

Improvement in weight-related measures (waist circumference, body fat percentage, visceral fat, ectopic fat deposits): The SURMOUNT-1 study treated overweight/obese patients with tirzepatide 5mg to 15 mg once weekly for 72 weeks showed an average waist circumference reduction of 14-18.5 cm, a body mass decrease of 33.9%, a lean mass reduction of 10.9%, and a decreased visceral fat of 40.1% compared to baseline^[85]^. Similar effects of semaglutide have also been observed in the STEP series^[76].^

Improvement in metabolic markers and physical and mental health: Both SURMOUNT and STEP series observed overall improvement in metabolic markers such as blood pressure, blood glucose, insulin, and blood lipid levels with the use of tirzepatide or semaglutide in overweight/obese patients^[76,85]^. They also resulted in significant improvements in self-reported physical health and mental health scores^[95]^.

Prevention of diabetes: it is predicted that tirzepatide (5-15 mg) was associated with a 16.0%-20.3% and 10.1%-11.3% reduction of developing diabetes in overweight/obese patients with prediabetes or normal blood glucose according to the SURMOUNT-1 study^[96]^.

Metabolic dysfunction-associated steatohepatitis (MASH): The SYNERGY-NASH study showed that 44% to 62% of overweight/obese patients with stage Ⅱ/Ⅲ fibrotic MASH treated with tirzepatide (5 to 15 mg) achieved complete remission without deterioration of liver fibrosis (compared to 10% in placebo group). Furthermore, 51% to 55% of these patients showed improvement of at least one phase fibrosis stage, with no cases of MASH worsening (30% in placebo group). Mean liver fat content decreased by 41.3% to 57.0% from baseline (9. 8% in placebo group)^[97]^. 36% to 59% of overweight/obese patients with stage Ⅰ ~ Ⅲ fibrosis treated with semaglutide for 72 weeks achieved complete remission without worsening hepatic fibrosis in a phase 2 clinical trial (17% in placebo group)^[98]^. The GLORY-1 study found that 63.3% to 73.2% of mean liver fat content decreased in overweight or obese patients (with liver fat content ≥5%) receiving mazdutide (4 to 6 mg) for 48 weeks (with an 8.2% increase in the placebo group)^[99]^.

Obstructive sleep apnea (OSA): The SURMOUNT-OSA study showed that treatment with tirzepatide (10 or 15 mg) significantly reduced apnea hypopnea index (AHI) in obese patients with moderate to severe OSA, achieving reduction of 27.4 events per hour for those without positive airway pressure (PAP) therapy and 30.4 events per hour for those with PAP therapy, respectively (compared to 4.8 and 6.0 events per hour in the placebo group, respectively)^[100]^.

Cardiorenal benefit: In the SELECT study, overweight/obese patients (non-diabetic) with cardiovascular disease were treated with semaglutide (2.4 mg once weekly) over an average of 34.2 months and follow-up of 39.8 months. Results indicated a 20% reduction in the risk of cardiovascular death, non-fatal myocardial infarction, or nonfatal stroke compared to the placebo group, as well as a 22% lower risk of chronic kidney disease or nephro-cause death than that of the placebo control group^[101]^. The post-hoc analysis of SURMOUNT-1 also showed that tirzepatide provided benefits in predicting cardiovascular disease risk^[102]^ and improving proteinuria^[103]^.

## 4.4 Adverse reactions of NuSH receptor agonists and therapeutic regimen

The new weight-loss drugs of NuSH class are mainly gastrointestinal reactions, including nausea, diarrhea, constipation, vomiting, dyspepsia, epigastric pain, and decreased appetite. These side effects are generally mild to moderate and most commonly occur during the initial treatment phase and dose escalation period. Over time, these reactions often diminish as patients adapt to the medication. To help manage gastrointestinal reactions, patients should be educated on dietary guidelines and the titration method, starting with small doses and gradually increasing them as they become accustomed. For patients experiencing gastrointestinal adverse effects during dose escalation, the following recommendations are suggested: (1) dietary modification (e.g., reduce food intake or switch to smaller, more frequent meals); (2) symptomatic drugs for gastrointestinal reactions (such as omeprazole and domperidone) can be considered if dietary modification is ineffective; (3) if symptoms persist, return to the previously tolerated dose, and consider increase the dose after the symptoms are relieved; (4) if the patient remains intolerant to the current dose, consider the previously dose as the maximum tolerated dose and maintain treatment.

Other potential adverse reactions include an increased risk of hypoglycemia when combined with sulfonylurea drugs or insulin, increased amylase and lipase levels, cholelithiasis, increased heart rate, allergic reactions, injection site reactions, and acute pancreatitis. However, the incidence of these reactions is low. It is important to note that patients with a history of pancreatitis need to be cautious with these drugs and this product should be discontinued immediately if pancreatitis is suspected. These drugs should not be used if pancreatitis is confirmed. For patients with elevated triglyceride levels (≥5.65 mmol/L), triglyceride-lowering therapy is recommended before initiating NuSH agonist for weight intervention due to the heightened risk of acute pancreatitis. Additionally, GLP-1RA has been associated with thyroid C-cell tumors in rodent studies ^[104]^. Although there is currently insufficient clinical evidence linking GLP-1RA to human thyroid C-cell tumors^[105-106]^, a personal or family history of medullary thyroid cancer or multiple endocrine neoplasia type 2 constitutes a contraindication for this class of drugs.

Additionally, for patients with malnutrition or sarcopenia, as well as patients who need long-term medication, it is worth noting that when using these new powerful weight loss drugs. Reasonable muscle-building exercises and high-protein diets should be emphasized to prevent muscle loss, as significant and substantial weight-loss will inevitably bring about a decrease in lean mass.

## 4.5 Monitoring and adjustment of weight-loss drugs in long-term management

Timely monitoring and adjustment of treatment regimens are essential during medication use to prevent loss of patient confidence due to therapeutic inertia. It is recommended that patients should receive guidance on diet, exercise, and psychological support alongside their medication instructions. Educating patients to self-monitor their weight and waist circumference weekly, and to record these measurements, can improve patient compliance. This can be facilitated by integrating wearable smart devices and other methods. Follow-up visits should be strengthened, with evaluations of drug efficacy and safety at least once monthly. After the first three months of treatment, the frequency of visits can be adjusted based on individual circumstances. Body fat, visceral fat, and metabolic indicators (such as blood glucose, insulin, blood pressure, and blood lipids) should be assessed every three to six months.

A treatment failure or a weight-loss plateau may be considered if a patient loss less than 5% of their weight over three consecutive months. It is important to communicate openly with the patient about their adherence to both lifestyle changes and medication. This dialogue will help reassess the weight-loss regimen. Increasing drug dose or upgrading to a more potent weight-loss drug may be considered if necessary.

## 4.6 Long-term medication for weight maintenance after weight loss

Weight maintenance after weight loss, particularly preventing rebound, is the most challenging part of long-term weight management. Weight rebound is defined as the change in weight gain over time from the peak of the lowest weight loss achieved during a weight-loss intervention, with baseline body weight gaining more than 3%-5% of the cut-off point for weight regain^[107]^. Weight loss is accompanied by changes in the metabolic adaptation, increased appetite, decreased satiety, and decreased metabolic rate, thus resisting sustained weight loss, leading to weight regain. Such rebound leads to worsening of obesity-related comorbidities, impairing patients' confidence in weight loss^[108-109]^. Given the chronic and relapsing nature of obesity, it is particularly important of long-term weight management as most weight-loss methods face the challenge of regain after cessation of the intervention.

For intensive treatment with weight-loss drugs, after the body weight reaches the target or the maximum weight loss of the individual, the commonly used clinical maintenance regimens include: regimen 1, maintains the original weight-loss drugs and lifestyle interventions; regimen 2, reduces the dose of weight-loss drugs or taking them intermittently, combined with lifestyle interventions; regimen 3, discontinuation of weight-loss drugs, simple lifestyle intervention. Evidence indicates that weight regain after intensive therapy with medication, when switched to lifestyle intervention alone, is significantly higher than in the continuous medication group. There is currently insufficient evidence regarding dose reduction or intermittent drug use. In the global phase 3 clinical studies SURMOUNT-4 and STEP-4, the efficacy of regimen 1 (maintenance medication with lifestyle intervention) was compared to regimen 3 (discontinuation of medication lifestyle intervention alone) after intensive treatment with weight-loss drugs. In SURMOUNT-4 study, overweight or obese subjects received a lifestyle intervention combined with tirzepatide at the maximum tolerated dose for 36 weeks (mean weight loss of 21.1%) and were assigned to continue treatment or switch to placebo for 52 weeks. Those who continued with tirzepatide further lost 6.7% of body weight, compared to those who switched to placebo regained 14.8% of body weight (lifestyle intervention only), with a 21.4% difference between the two groups^[1]^. In STEP-4 study, participants who continued semaglutide treatment for one year lost an additional 8.8% of their body weight, while those who switched to placebo (lifestyle intervention only) regained 6.5% of their weight, with a 15.3% difference between the two groups after an initiation of semaglutide treatment for 20 weeks achieved a 10.6% weight loss^[110]^.

In addition, studies have demonstrated that long-term use of weight-loss drugs can yield sustained benefits. In the SELECT study, which was followed for an average of 34.2 months and followed up for 39.8 months in overweight/obese patients with cardiovascular disease, semaglutide 2.4 mg once weekly effectively maintained their weight loss throughout the treatment period. Participants achieved a mean weight loss of 9.39% compared to 0.88% in the placebo control. Furthermore, the risk of a composite outcome of cardiovascular death, nonfatal myocardial infarction, or nonfatal stroke was reduced by 20% compared to placebo. The risk of developing chronic kidney disease or nephro-cause death was 22% lower than that of placebo controls, and the risk of developing prediabetes was reduced by 67% among subjects with normal baseline glucose tolerance. A higher proportion of patients with prediabetes achieved euglycemia, along with sustained benefits in blood pressure and lipids levels. However, the rate of adverse reactions (mainly gastrointestinal reactions) leading to drug discontinuation was 16.6%, significantly higher than the 8.2% seen in the placebo group^[101]^.

In summary, based on the available evidence, it is advisable to maintain the original weight-loss medications and lifestyle interventions as consistently as possible during the maintenance treatment period following intensive therapy. However, appropriate adjustments should be made according to the individual circumstances (such as economic factors and adherence levels). Patient compliance is particularly important. The maintenance plan should be fully communicated with the patient, emphasizing the importance of a regimen that is acceptable and sustainable for the long term. Education during the maintenance phase is vital; patients should be fully informed about the likelihood of weight regain after stopping interventions, the significance of long-term weight stability, and the purpose and methods of maintenance treatment, including self-monitoring and follow-up plans. Ensuring patient understanding and cooperation is essential for successful long-term weight maintenance.

## 4.7 Combined application of weight-loss drugs

Different types of weight-loss drugs may have different mechanisms, and the combination of these drugs may have an additive or synergistic effect^[111]^. It is essential to address the clinical need for combination therapies to effectively manage weight at target levels. However, there is currently a lack of robust evidence-based data regarding combination drug therapy. In addition to approved weight-loss medications, including metformin, sodium-glucose cotransporter 2 inhibitors (SGLTi) and α-glycosidase inhibitors, certain proprietary Chinese medicines, while not specifically designed for weight-loss, may also have a certain weight-loss effect. If a single bariatric medication proves ineffective, a combination approach may be necessary. It is advisable to select drugs from different classes that have complementary mechanisms of action. Off-label use of medications presents regulatory challenges and should only be implemented after appropriate procedures have been followed in accordance with relevant regulations. Alongside monitoring efficacy, it is crucial to closely observe any adverse reactions and ensure compliance with off-label drug management protocols.

# 5. Medications for common comorbidities of obesity

Treating obesity can improve various comorbidities, as obesity is closely associated with a variety of systemic diseases. When selecting weight-loss medications, it's essential to take these comorbidities into account. Medications for managing comorbid conditions should be integrated into the comprehensive obesity treatment regimen, while the use of weight-increasing drugs should be minimized whenever possible. Table 4 lists the medication recommendations for common obesity-related comorbidities for reference.

**Table 4** Medication recommendations for common obesity comorbidities.

| Common comorbidities | Epidemiology | Recommended medication for comorbidities |
| --- | --- | --- |
| Impaired glucose tolerance | The prevalence of prediabetes in overweight and obese patients in China was 40.7% and 43.6%, respectively (32.6% in the normal weight population)^[112]^. | Most NuSH drugs have function of reducing glucose and weight.  Some hypoglycemic drugs also have the effect of weight loss, such as SGLT2is and metformin, which can be used in combination with weight loss drugs when necessary. |
| T2DM | The prevalence of diabetes in overweight and obese patients in China was 15.4% and 21.1%, respectively (7.8% in the normal weight population)^[112]^. |  |
| Hypertension | The prevalence of hypertension in overweight and obese patients in China was 20.7% and 36.9%, respectively (11.2% in normal weight population)^[9]^. | Most NuSH drugs are both antihypertensive and weight loss. Angiotensin II receptor antagonists (ARB) or angiotensin converting enzyme inhibitors (ACEI) can be the preferred choice. Calcium antagonists can be used as alternative drugs to control blood pressure because of their neutral effects on body weight. |
| Hyperlipemia | The prevalence of lipid disorders in overweight and obese patients in China was 42.4% and 31.3%, respectively (16.9% in normal weight population)^[9,113]^. | Statins are recommended for patients with elevated cholesterol, and fibrates are recommended for patients with elevated triglycerides.  For patients who are intolerance with the above drugs, ezetimibe and PCSK 9 inhibitors can be considered if necessary. |
| MASH | The prevalence of MASH in overweight and obese patients in China was 81.8% and 49.0%, respectively (13.3% in normal weight population)^[9]^. | NuSH with evidence of liver benefit, such as tirzepatide and semaglutide) are referenced.  Symptomatic treatment including vitamin E, silymarin, bicyclic alcohol, polyene phosphatidylcholine, or glutathione, ursodeoxycholic acid, etc for MASH or progressive liver fibrosis and other liver disease or liver transaminase elevated. |
| OSA | The prevalence was 40% to 90%^[114]^. | NuSH (such as tirzepatide) is recommended to be used.  Continuous positive pressure ventilation for symptomatic treatment if necessary. |
| PCOS and sterility | The prevalence of PCOS in childbearing women with obesity in China was 67%^[115]^. | Comprehensive therapy is mainly for weight loss and androgen reduction.  NuSH drugs can be combined with metformin and thiazolidinediones to improve insulin resistance.  Androgen antagonism and ovulation induction therapy if necessary. |
| Cardiovascular disease | Compared with normal weight, overweight men and women had 21% and 32% increased risk, obese men and women had 67% and 85% increased risk.  11.98% of CVD deaths in China were attributed to high BMI in 2019^[116]^. | NuSH drugs with evidence of cardiovascular benefit (e.g., tirzepatide and semaglutide).  SGLT2i with cardiovascular disease indications (e.g., emagliflozin and dapagliflozin).  Other symptomatic treatments with cardiovascular benefit, including antiplatelet, lipid modulation, RASS inhibitors, diuresis, etc., according to the individual condition. |
| Hyperuricemia | The prevalence of hyperuricemia in overweight and obese patients in China was 21.1% and 12.8%, respectively (6.3% in the normal weight population)^[117-118]^ | When patients have gouty arthritis or urinary stones or potential risks, uric acid-lowering drugs such as allopurinol, febuxostat, benzbromarone, etc. can been used.  General adverse reactions and precautions of these drugs need to be considered. |
| Chronic kidney disease | The prevalence of chronic kidney disease in overweight and obese patients in China was 4.1% and 6.3%, respectively (3.7% in the normal weight population)^[119]^. | NuSH drugs with evidence of renal protection (e. g., tirzepatide and semaglutide)) and SGLT2i (e.g., emagliflozin and dapagliflozin).  RAAS inhibitors with indications for chronic kidney disease can also be effective in the short term when necessary. |
| Mood disorder | The prevalence of depression in overweight and obese women is 32.1% and 29.5%, and in overweight and obese men is 17.7% and 16.2%, respectively^[120]^ | Patients with depression and other mood disorders need to pay attention to the influence of antidepressants on weight.  Weight loss or less weight influencing drugs can be considered under the premise of controlled mood symptoms (such as fluoxetine, sertraline, bupropion) and not priority with weight gain drugs (such as paroxetine, amitriptyline, mirtazapine) ^[65,121]^.  It is noted that some mood disorders patients may use the weight effects of antipsychotics such as thioridazazine, clozapine, olanzapine, risperidone (average weight gain 2.1~4. 5 kg).  Commonly used drugs that gain more than 7% of weight also include quintiapine, haloperidol, and trifluoperazine and other ^[122]^.  Discussing weight problems and the effects of drugs on body weight with psychiatrist to develop a management plan is recommended. |

MASH: metabolic dysfunction-associated steatohepatitis; NuSH: nutrient-stimulated hormone; T2DM: type 2 diabetes; ARB: angiotensin II receptor antagonists；ACEI: angiotensin converting enzyme inhibitors; PCSK 9; PCOS: polycystic ovary syndrome; OSA: obstructive sleep apnea; CVD: cardiovascular disease; BMI: body mass index.

# 6. Treatment of special populations

## 6.1 Childhood Obesity

In recent years, the incidence of obesity among children has risen significantly, with global data from 2020 indicating that China has the highest increase in BMI among children worldwide. The latest national survey data reveals that the prevalence of childhood obesity and overweight in China is as high as 24.7%, with higher rates observed in northern regions compared to the south, and in boys compared to girls^[123]^. Obesity can cause a variety of physical, psychological and social issues, and 75%-80% of children with obesity may continue to experience these challenges into adulthood, significantly impacting their health and longevity^[124]^.

The preferred approach for weight loss in overweight or obese children is lifestyle intervention, given the unique considerations of their growth and development. The general principle is ensuring their nutritional needs to support normal growth and development. A dietary strategy emphasizing low fat, low sugar, low salt, high protein, and moderate fiber intake is recommended. It is necessary to minimize additional meals and reduce fast food consumption, promote mindful eating, and limit screen time during meals. It is recommended that children and adolescents over the age of 6 insist on more than 60 minutes of moderate-to-high-intensity exercise daily, along with reducing screen time and increasing household activities. Effective management of childhood obesity requires collaboration among schools, families, and healthcare providers, alongside psychological support to combat obesity-related stigma^[125-126]^.

Drug therapy is recommended only for obese children or adolescents who are struggled to manage weight gain or improve comorbidities despite intensive lifestyle changes. Weight-loss drugs are not recommended for overweight children or adolescents^[127]^. Drugs currently approved for obese children or adolescents aged 12 years old and older in the United States include orlistat (120 mg), liraglutide (3.0 mg), semaglutide (2.4 mg), and phentermine/topiramate^[14]^. Although no weight-loss medications are currently approved for children in China, clinical trials are ongoing. For children or adolescents with moderate to severe obesity and comorbidities who have not responded to lifestyle interventions, weight-loss drugs may be considered after thorough discussions with guardians and obtaining informed consent. It is recommended to start with a low dose, not exceeding the adult tolerance, with closely monitoring for side effects. For children with obesity-related comorbidities, symptomatic treatment should be provided based on weight loss.

## 6.2 Gestational obesity

The prevalence of overweight and obesity among women of childbearing age in China has reached 25.4% and 9.2%, respectively, with only 17.1% actively managing their weight^[128]^. Obese women often have irregular menstrual cycles, anovulation, and conditions such as polycystic ovary syndrome (PCOS), which increase their risk of infertility. In addition, obesity during pregnancy increases the likelihood of maternal and fetal perinatal complications, with potential long-term effects on the offspring^[129]^. Therefore, it is crucial for obese women to actively engage in weight management before, during, and after pregnancy, with specific drug recommendations at each stage.

Before pregnancy, weight management strategies mirror those for the general adult population and may include intensive lifestyle interventions, medications, and bariatric surgery. Given the time required for pregnancy and the effect of lifestyle interventions is limiting, early medication is recommended to achieve target weight goals to provide conditions for pregnancy. NuSH receptor agonists or orlistat are recommend. NuSH receptor agonists can improve ovarian function and fertility, especially in women with PCOS^[130]^. Orlistat had no effect on fertility. Metformin can also aid in weight management for obese women with diabetes by improving insulin sensitivity and increase the chance of natural pregnancy. Metformin is not routinely recommended for weight control in non-diabetic obese women, but can be considered as an adjunct in patients with severe insulin resistance since there is no evidence that it can directly improve maternal and infant outcomes^[3]^. Drug therapy should be discontinued if pregnancy is planned or existed during treatment.

During pregnancy, weight management primarily involves dietary and exercise interventions. The use of medications such as NuSH receptor agonists, orlistat, and metformin is not recommended to avoid potential fetal harm^[8, 10-11]^.

Postpartum, excessive weight retention refers to the inability of women to return to the pre-pregnancy level after childbirth. Excessive weight gain before and during pregnancy is the main cause of postpartum weight retention. Postpartum weight retention is a potential risk factor for women to develop overweight and obesity in the short and long term. Strategies to prevent postpartum weight retention include a healthy diet, early exercise, breastfeeding for at least six months, social support, and measurements to prevent postpartum depression. Weight-loss medications are not recommended during lactation but can be considered after breastfeeding ends, following the same guidelines as for the general adult population.

## 6.3 The elderly

The benefits and risks of weight management in the elderly are controversial. The elderly often have multiple comorbidities, such as hypertension, hyperglycemia and hyperlipidemia, along with cardiovascular disease and hepatic and renal insufficiency. Malnutrition and sarcopenic obesity are also prevalent in this population. The elderly also tend to have more drugs in common^[131]^. In addition, obesity often takes a long time to present adverse outcomes, and a certain amount of fat reserves may have significance in fighting severe diseases. Therefore, weight management in the elderly should therefore be approached cautiously, focusing on gradual weight loss and preventing malnutrition and sarcopenia. The benefit of other comorbidities, particularly cardiovascular and renal comorbidities can be considered^[7]^. Lifestyle interventions should emphasize nutrient-dense foods and nutritional support. Exercise regimens should include a combination of aerobic and resistance training to maintain endurance and minimize the loss of lean mass^[7]^. When considering pharmacological options, it is important to choose medications that are simple and have high adherence rates, starting with low doses and adjusting as needed while monitoring for adverse effects.

The effect of weight loss by orlistat is weak. The tolerance of gastrointestinal adverse effects (such as steatorrhea and vitamin deficiency) in the elderly need to be considered and the coping methods need to be fully informed. In addition, it is necessary to pay attention to the therapeutic effect and drug concentration when orlistat taken with antiepileptic drugs, amiodarone, cyclosporine, levothyroxine, warfarin, and antiviral drugs^[1]^. NuSH receptor agonists demonstrate significant weight-loss effects and are relatively safe for the elderly^[2]^. Furthermore, most NuSH receptor agonist improved blood glucose, blood pressure, and lipids levels, and some NuSH receptor agonists (such as semaglutide and tirzepatide) improved cardiovascular and renal outcomes. Therefore, they are recommended for weight management in the elderly. It is necessary to reasonably control the weight loss rate when taking medication, avoid the occurrence and development of malnutrition and sarcopenia due to its powerful weight loss effect, and pay attention to gastrointestinal adverse reactions.

## 6.4 Patients with liver or kidney insufficiency

In obese patients with liver or kidney dysfunction, identifying the underlying cause is critical. Weight loss can help restore liver enzyme levels and reduce proteinuria in case of obesity-related fatty liver disease and nephropathy. For liver and kidney dysfunction stemming from other causes, a careful assessment of the benefits and risks of weight loss is essential. Life expectancy needs to be prioritized before the benefits of weight loss and medications use, especially for those with severe liver and kidney dysfunction^[23]^.

Weight-loss drug for patients with hepatic and renal dysfunction: (1) Tirzepatide can be used without dose adjustment in patients with hepatic dysfunction and renal dysfunction, including end-stage renal disease. There is limited experience in patients with severe impaired renal function and end-stage renal disease and severe impaired liver function. (2) Semaglutide does not require dose adjustments in patients with mild, moderate, and severe renal dysfunction, but experience with end-stage renal disease is limited; dose adjustment is not required in patients with mild to moderate hepatic dysfunction, but experience with severe hepatic dysfunction is limited. (3) Liraglutide is not require dose adjustment in patients with mild renal dysfunction, has limited experience in the treatment of patients with moderate renal dysfunction, and is not recommended for patients with severe renal dysfunction, including patients with end-stage renal disease; experience in the treatment of patients with hepatic dysfunction is limited and is not recommended for patients with mild, moderate, or severe hepatic dysfunction. (4) Orlistat does not need dose adjustment in patients with hepatic and renal dysfunction, but it is contraindicated in patients with cholestasis.

## 6.5 Perioperative of metabolic surgery

For patients with severe obesity unresponsive to conservative management, surgical options may be discussed. The use of perioperative weight-loss drugs can help reduce the surgical risks and prevent postoperative weight gain.

Drugs such as NuSH agonists may be beneficial for addressing obesity-related lung disease (e.g., hypoxemia, OSA), reducing intra-abdominal fat, minimizing anesthesia and surgical risks. However, these medications should be discontinued two weeks prior to surgery to prevent complications^[132]^.

About 25% of patients experience insufficient weight loss or postoperative weight regain after metabolic surgery. Postoperative bariatric drug intervention may benefit these patients^[133-137]^. A multidisciplinary evaluation is recommended, considering factors such as diet, psychology, and surgical history, before initiating pharmacological treatment. NuSH receptor agonists have a positive effect on postoperative weight loss as a preferred option^[136-138]^. Orlistat has a limited effect and can be used as an option^[133-134]^. Furthermore, the choice of medication should align with the bariatric surgical procedure. If orlistat is combined with LSG, it can improve the symptoms of postoperative constipation. However, this drug should not be used for patients who are prone to diarrhea after diversion surgery. In addition to the use of glycosidase inhibitors, NuSH agonists help maintain blood glucose homeostasis and improve hypoglycemia symptoms for patients with postoperative dumping syndrome^[139]^. Genetic testing is recommended for patients with no effective weigh loss if necessary and targeted therapy can be used if hereditary obesity is identified.

## 6.6 Obesity syndrome

Obesity syndromes include obesity syndromes associated with neurodevelopmental disorders as well as monogenic obesity syndromes, which account for about 5% of all severe obesity cases^[140]^. With the development of genetic testing technology, an individualized treatment plan is provided for syndromic obesity. The medication for obesity syndrome is also different from that of simple obesity.

Setmelanotide is an MC4R agonist currently approved by the FDA for targeting genetic defects such as POMC, PCSK1, and LEPR or for Bardet-Biedl syndrome confirmed by genetic testing. After one year of treatment with setmelanotide, about 80% of obese patients with POMC or PCSK1 deficiency lost more than 10% of body weight，45.5% of obese patients with leptin receptor-deficient lost more than 10% of their body weight^[65, 130, 141]^.

Metreleptin is a recombinant human leptin analogue that mimics the physiological effects of leptin by binding to and activating leptin receptor. It is currently approved as an alternative treatment for patients with congenital or acquired lipodystrophy and anorexia nervosa^[57, 142]^.

It needs be noted that none of the above drugs are recommended for use in obese patients with non-specific gene mutations, and none of them are currently approved in China.

**Steering Committee (in alphabetical order):** Chen Lulu (Union Hospital, Tongji Medical College, Huazhong University of Science and Technology), Guo Lixin (Beijing Hospital, National Geriatrics Center), Mu Yiming (Chinese People's Liberation Army General Hospital), Wang Weiqing (Ruijin Hospital, Shanghai Jiao Tong University School of Medicine), Yan Li (Sun Yat-sen Memorial Hospital, Sun Yat-sen University), Zhou Zhiguang (Second Xiangya Hospital, Central South University), Zou Dajin (Tongren Hospital, Shanghai Jiao Tong University).

**Contact:** Jiajun Zhao (Provincial Hospital Affiliated to Shandong First Medical University) , Ji Linong (Peking University People's Hospital), Qu Shen (The Tenth People's Hospital of Tongji University)

**Writers:** Qu Shen (The Tenth People's Hospital of Tongji University), Song Yongfeng (The Affiliated Central Hospital of Shandong First Medical University), Cai Xiaoling (Peking University People's Hospital), Li Sheyu (West China Hospital, Sichuan University).

**Committee members (in alphabetical order):** Bao Yuqian (The Sixth People's Hospital Affiliated to Shanghai Jiao Tong University), Bi Yan (Drum Tower Hospital Affiliated to Nanjing University), Bi Yufang (Ruijin Hospital Affiliated to Shanghai Jiao Tong University School of Medicine), Cai Xiaoling (Peking University People's Hospital), Chen Haibing (The Tenth People's Hospital Affiliated to Tongji University), Chen Hong (Zhujiang Hospital of Southern Medical University), Chen Liming (Zhu Xianyi Memorial Hospital of Tianjin Medical University), Fu Junfen (Children's Hospital Affiliated to Zhejiang University School of Medicine), Li Sheyu (West China Hospital, Sichuan University), Li Xiaoying (Huashan Hospital, Fudan University), Li Yong (Huashan Hospital, Fudan University), Chao Liu (Jiangsu Provincial Hospital of Integrated Traditional Chinese and Western Medicine), Lu Hao (Shuguang Hospital, Shanghai University of Traditional Chinese Medicine), Li Li (The First Affiliated Hospital of Ningbo University), Ma Jing (Renji Hospital, Shanghai Jiao Tong University School of Medicine), Qu Shen (The Tenth People's Hospital of Tongji University), Song Yongfeng (Provincial Hospital of Shandong University), Tong Nanwei (West China Hospital, Sichuan University), Wang Guang (Beijing Chaoyang Hospital, Capital Medical University), Jiqiu Wang (Ruijin Hospital, Shanghai Jiao Tong University School of Medicine), Wei Lai (Beijing Tsinghua Changgung Hospital, Tsinghua University), Yu Xuefeng (Union Hospital, Tongji Medical College, Huazhong University of Science and Technology), Yuan Huijuan (Henan Provincial People's Hospital) , Mingxia Yuan (Beijing Friendship Hospital, Capital Medical University), Huijie Zhang (Nanfang Hospital, Southern Medical University), Hongting Zheng (Xinqiao Hospital, Third Military Medical University), Zhiming Zhu (Chongqing Daping Hospital), Zhao Jiajun (Provincial Hospital Affiliated to Shandong First Medical University), Ji Linong (Peking University People's Hospital), Zeng Tianshu (Union Hospital Affiliated to Tongji Medical College, Huazhong University of Science and Technology).

**References**

1. Federation WO. World Obesity Atlas 2024 [M/OL]. 2024 March://www.worldobesity.org/.

2. WANG L, ZHOU B, ZHAO Z, et al. Body-mass index and obesity in urban and rural China: findings from consecutive nationally representative surveys during 2004-18 [J]. Lancet (London, England), 2021, 398(10294): 53-63.DOI:10.1016/s0140-6736(21)00798-4

3. PAN X F, WANG L, PAN A. Epidemiology and determinants of obesity in China [J]. The lancet Diabetes & endocrinology, 2021, 9(6): 373-92.DOI:10.1016/s2213-8587(21)00045-0

4. YANG XL, ZHANG B, ZHANG JG, et al. The Trend of Waist Circumference Distribution in Adults from Nine Provinces in China from 1993 to 2011 [J]. Journal of Nutrition, 2014, 36(03): 212-7. DOI:10.13325/j.cnki.acta.nutr.sin.2014.03.003

5. Ludwig DS, Ebbeling CB. The carbohydrate-insulin model of obesity: Beyond "calories in, calories out."[J]. JAMA Internal Medicine, 2018, 178(8), 1098-1103. doi:10.1001/jamainternmed.2018.2933.

6. BRAY G A, KIM K K, WILDING J P H. Obesity: a chronic relapsing progressive disease process. A position statement of the World Obesity Federation [J]. Obes Rev, 2017, 18(7): 715-23.DOI:10.1111/obr.12551

7. APOVIAN, C.M. Obesity: Definition, comorbidities, causes, and burden[J]. American Journal of Managed Care, 2016, 22(7 Suppl), S176-S185.

8. KIVIMäKI M, STRANDBERG T, PENTTI J, et al. Body-mass index and risk of obesity-related complex multimorbidity: an observational multicohort study [J]. The lancet Diabetes & endocrinology, 2022, 10(4): 253-63.DOI:10.1016/s2213-8587(22)00033-x

9. CHEN K, SHEN Z, GU W, et al. Prevalence of obesity and associated complications in China: A cross-sectional, real-world study in 15.8 million adults [J]. Diabetes Obes Metab, 2023, 25(11): 3390-9.DOI:10.1111/dom.15238

10. LI J, SHI Q, GAO Q, et al. Obesity pandemic in China: epidemiology, burden, challenges, and opportunities [J]. Chinese medical journal, 2022, 135(11): 1328-30.DOI:10.1097/cm9.0000000000002189

11. Peeters A, Barendregt JJ, Willekens F, et al. Obesity in adulthood and its consequences for life expectancy: A life-table analysis. Annals of Internal Medicine, 2023, 138(1), 24-32. doi:10.7326/0003-4819-138-1-200301070-00008.

12. ZENG Q, LI N, PAN X F, et al. Clinical management and treatment of obesity in China [J]. The lancet Diabetes & endocrinology, 2021, 9(6): 393-405.DOI:10.1016/s2213-8587(21)00047-4

13. QIN X, PAN J. The Medical Cost Attributable to Obesity and Overweight in China: Estimation Based on Longitudinal Surveys [J]. Health economics, 2016, 25(10): 1291-311.DOI:10.1002/hec.3217

14. WANG Y, ZHAO L, GAO L, et al. Health policy and public health implications of obesity in China [J]. The lancet Diabetes & endocrinology, 2021, 9(7): 446-61.DOI:10.1016/s2213-8587(21)00118-2

15. LITWIN M, KUŁAGA Z. Obesity, metabolic syndrome, and primary hypertension [J]. Pediatric nephrology (Berlin, Germany), 2021, 36(4): 825-37.DOI:10.1007/s00467-020-04579-3

16. MECHANICK J I, HURLEY D L, GARVEY W T. ADIPOSITY-BASED CHRONIC DISEASE AS A NEW DIAGNOSTIC TERM: THE AMERICAN ASSOCIATION OF CLINICAL ENDOCRINOLOGISTS AND AMERICAN COLLEGE OF ENDOCRINOLOGY POSITION STATEMENT [J]. Endocrine practice: official journal of the American College of Endocrinology and the American Association of Clinical Endocrinologists, 2017, 23(3): 372-8.DOI:10.4158/ep161688.Ps

17. ACOSTA A, CAMILLERI M, ABU DAYYEH B, et al. Selection of Antiobesity Medications Based on Phenotypes Enhances Weight Loss: A Pragmatic Trial in an Obesity Clinic [J]. Obesity (Silver Spring, Md), 2021, 29(4): 662-71.DOI:10.1002/oby.23120

18. LIN Z, FENG W, LIU Y, et al. Machine Learning to Identify Metabolic Subtypes of Obesity: A Multi-Center Study [J]. Frontiers in endocrinology, 2021, 12(713592.DOI:10.3389/fendo.2021.713592

19. LIU Y, SHENG C, FENG W, et al. A multi-center study on glucometabolic response to bariatric surgery for different subtypes of obesity [J]. Frontiers in endocrinology, 2022, 13(989202.DOI:10.3389/fendo.2022.989202

20. LEAN M E, LESLIE W S, BARNES A C, et al. Primary care-led weight management for remission of type 2 diabetes (DiRECT): an open-label, cluster-randomised trial [J]. Lancet (London, England), 2018, 391(10120): 541-51.DOI:10.1016/s0140-6736(17)33102-1

21. ERRATUM. Association of Weight Loss Maintenance and Weight Regain on 4-Year Changes in CVD Risk Factors: the Action for Health in Diabetes (Look AHEAD) Clinical Trial. Diabetes Care 2016;39: 1345-1355 [J]. Diabetes Care, 2016, 39(12): 2318.DOI:10.2337/dc16-er12

22. HALL K D, GUO J. Obesity Energetics: Body Weight Regulation and the Effects of Diet Composition [J]. Gastroenterology, 2017, 152(7): 1718-27.e3. DOI:10.1053/j.gastro.2017.01.052

23. HASLAM D W, JAMES W P. Obesity [J]. Lancet (London, England), 2005, 366(9492): 1197-209.DOI:10.1016/s0140-6736(05)67483-1

24. SCHETZ M, DE JONG A, DEANE A M, et al. Obesity in the critically ill: a narrative review [J]. Intensive care medicine, 2019, 45(6): 757-69.DOI:10.1007/s00134-019-05594-1

25. GLOBAL B M I M C, DI ANGELANTONIO E, BHUPATHIRAJU SH N, et al. Body-mass index and all-cause mortality: individual-participant-data meta-analysis of 239 prospective studies in four continents [J]. Lancet (London, England), 2016, 388(10046): 776-86.DOI:10.1016/s0140-6736(16)30175-1

26. DIXON J B, CHUANG L M, CHONG K, et al. Predicting the glycemic response to gastric bypass surgery in patients with type 2 diabetes [J]. Diabetes Care, 2013, 36(1): 20-6.DOI:10.2337/dc12-0779

27. STEVEN S, HOLLINGSWORTH K G, AL-MRABEH A, et al. Very Low-Calorie Diet and 6 Months of Weight Stability in Type 2 Diabetes: Pathophysiological Changes in Responders and Nonresponders [J]. Diabetes Care, 2016, 39(5): 808-15.DOI:10.2337/dc15-1942

28. GADDE K M, MARTIN C K, BERTHOUD H R, et al. Obesity: Pathophysiology and Management [J]. J Am Coll Cardiol, 2018, 71(1): 69-84. DOI:10.1016/j.jacc.2017.11.011

29. ELMALEH-SACHS A, SCHWARTZ J L, BRAMANTE C T, et al. Obesity Management in Adults: A Review [J]. JAMA, 2023, 330(20): 2000-15.DOI:10.1001/jama.2023.19897

30. FRANZ M J, VANWORMER J J, CRAIN A L, et al. Weight-loss outcomes: a systematic review and meta-analysis of weight-loss clinical trials with a minimum 1-year follow-up [J]. Journal of the American Dietetic Association, 2007, 107(10): 1755-67. DOI:10.1016/j.jada.2007.07.017

31. JENSEN M D, RYAN D H, APOVIAN C M, et al. 2013 AHA/ACC/TOS guideline for the management of overweight and obesity in adults: a report of the American College of Cardiology/American Heart Association Task Force on Practice Guidelines and The Obesity Society [J]. Circulation, 2014, 129(25 Suppl 2): S102-38.DOI:10.1161/01.cir.0000437739.71477.ee

32. CAZZOLA R, RONDANELLI M, TROTTI R, et al. Effects of weight loss on erythrocyte membrane composition and fluidity in overweight and moderately obese women [J]. The Journal of nutritional biochemistry, 2011, 22(4): 388-92. DOI:10.1016/j.jnutbio.2010.03.007

33. CHAE J S, PAIK J K, KANG R, et al. Mild weight loss reduces inflammatory cytokines, leukocyte count, and oxidative stress in overweight and moderately obese participants treated for 3 years with dietary modification [J]. Nutrition research (New York, NY), 2013, 33(3): 195-203. DOI:10.1016/j.nutres.2013.01.005

34. RODRIGUEZ A J, SCOTT D, EBELING P. Effect of weight loss induced by energy restriction on measures of arterial compliance: A systematic review and meta-analysis [J]. Atherosclerosis, 2016, 252(201-2. DOI:10.1016/j.atherosclerosis.2016.06.043

35. BRAY G A, HEISEL W E, AFSHIN A, et al. The Science of Obesity Management: An Endocrine Society Scientific Statement [J]. Endocrine reviews, 2018, 39(2): 79-132.DOI:10.1210/er.2017-00253

36. Chinese Medical Association Nutrition and Metabolism Management Branch, the Clinical Nutrition Branch of the Chinese Medical Association, the Diabetes Branch of the Chinese Medical Association, et al. Chinese Guidelines for Medical Nutritional Therapy of Overweight/Obesity (2021) [J]. Chinese Journal of Medical Frontiers (Electronic Edition), 2021, 13(11), 1-55. doi: 10.12037/YXQY.2021.11-01.

37. LARSEN R N, MANN N J, MACLEAN E, et al. The effect of high-protein, low-carbohydrate diets in the treatment of type 2 diabetes: a 12 month randomised controlled trial [J]. Diabetologia, 2011, 54(4): 731-40.DOI:10.1007/s00125-010-2027-y

38. TOBIAS D K, CHEN M, MANSON J E, et al. Effect of low-fat diet interventions versus other diet interventions on long-term weight change in adults: a systematic review and meta-analysis [J]. Lancet Diabetes Endocrinol, 2015, 3(12): 968-79.DOI:10.1016/s2213-8587(15)00367-8

39. ZHONG W, WANG H, YANG Y, et al. High-protein diet prevents fat mass increase after dieting by counteracting Lactobacillus-enhanced lipid absorption [J]. Nature metabolism, 2022, 4(12): 1713-31.DOI:10.1038/s42255-022-00687-6

40. VAN BAAK M A, MARIMAN E C M. Obesity-induced and weight-loss-induced physiological factors affecting weight regain [J]. Nature reviews Endocrinology, 2023, 19(11): 655-70.DOI:10.1038/s41574-023-00887-4

41. VARADY K A, CIENFUEGOS S, EZPELETA M, et al. Clinical application of intermittent fasting for weight loss: progress and future directions [J]. Nature reviews Endocrinology, 2022, 18(5): 309-21.DOI:10.1038/s41574-022-00638-x

42. ESPOSITO K, MAIORINO M I, CIOTOLA M, et al. Effects of a Mediterranean-style diet on the need for antihyperglycemic drug therapy in patients with newly diagnosed type 2 diabetes: a randomized trial [J]. Annals of internal medicine, 2009, 151(5): 306-14.DOI:10.7326/0003-4819-151-5-200909010-00004

43. NORDMANN A J, SUTER-ZIMMERMANN K, BUCHER H C, et al. Meta-analysis comparing Mediterranean to low-fat diets for modification of cardiovascular risk factors [J]. The American journal of medicine, 2011, 124(9): 841-51.e2. DOI:10.1016/j.amjmed.2011.04.024

44. SHENOY S F, POSTON W S, REEVES R S, et al. Weight loss in individuals with metabolic syndrome given DASH diet counseling when provided a low sodium vegetable juice: a randomized controlled trial [J]. Nutrition journal, 2010, 9(8.DOI:10.1186/1475-2891-9-8

45. CHAO AM, QUIGLEY KM, WADDEN TA. Dietary interventions for obesity: clinical and mechanistic findings [J]. J Clin Invest, 2021, 131(1): e140065. DOI: 10.11712/JCI140065.

46. MOON J, KOH G. Clinical evidence and mechanisms of high-protein diet-induced weight loss [J]. J Obes Metab Syndr, 2020, 29(3): 166-173. DOI: 10.7570/jomes20028.

47. CHIN S H, KAHATHUDUWA C N, BINKS M. Physical activity and obesity: what we know and what we need to know [J]. Obes Rev, 2016, 17(12): 1226-44.DOI:10.1111/obr.12460

48. FOSTER-SCHUBERT K E, ALFANO C M, DUGGAN C R, et al. Effect of diet and exercise, alone or combined, on weight and body composition in overweight-to-obese postmenopausal women [J]. Obesity (Silver Spring, Md), 2012, 20(8): 1628-38.DOI:10.1038/oby.2011.76

49. BEUTEL M E, DIPPEL A, SZCZEPANSKI M, et al. Mid-term effectiveness of behavioral and psychodynamic inpatient treatments of severe obesity based on a randomized study [J]. Psychotherapy and psychosomatics, 2006, 75(6): 337-45.DOI:10.1159/000095439

50. GUDZUNE K A, DOSHI R S, MEHTA A K, et al. Efficacy of commercial weight-loss programs: an updated systematic review [J]. Ann Intern Med, 2015, 162(7): 501-12.DOI:10.7326/m14-2238

51. ALAMUDDIN N, WADDEN T A. Behavioral Treatment of the Patient with Obesity [J]. Endocrinology and metabolism clinics of North America, 2016, 45(3): 565-80. DOI:10.1016/j.ecl.2016.04.008

52. TCHANG B G, SAUNDERS K H, IGEL L I. Best Practices in the Management of Overweight and Obesity [J]. The Medical clinics of North America, 2021, 105(1): 149-74. DOI:10.1016/j.mcna.2020.08.018

53. WADDEN T A, WEBB V L, MORAN C H, et al. Lifestyle modification for obesity: new developments in diet, physical activity, and behavior therapy [J]. Circulation, 2012, 125(9): 1157-70.DOI:10.1161/circulationaha.111.039453

54. MAZEREEL V, DETRAUX J, VANCAMPFORT D, et al. Impact of Psychotropic Medication Effects on Obesity and the Metabolic Syndrome in People With Serious Mental Illness [J]. Frontiers in endocrinology, 2020, 11(573479.DOI:10.3389/fendo.2020.573479

55. SERRETTI A, MANDELLI L. Antidepressants and body weight: a comprehensive review and meta-analysis [J]. The Journal of clinical psychiatry, 2010, 71(10): 1259-72. DOI:10.4088/JCP.09r05346blu

56. AVILA, C., HOLLOWAY, A.C., HAHN, M.K., et al.An overview of links between obesity and mental health. Current Obesity Reports, 2015, 4(3), 303-310. doi:10.1007/s13679-015-0164-9.

57. PERDOMO C M, COHEN R V, SUMITHRAN P, et al. Contemporary medical, device, and surgical therapies for obesity in adults [J]. Lancet (London, England), 2023, 401(10382): 1116-30.DOI:10.1016/s0140-6736(22)02403-5

58. MüLLER T D, BLüHER M, TSCHöP M H, et al. Anti-obesity drug discovery: advances and challenges [J]. Nature reviews Drug discovery, 2022, 21(3): 201-23.DOI:10.1038/s41573-021-00337-8

59. BESSESEN D H, VAN GAAL L F. Progress and challenges in anti-obesity pharmacotherapy [J]. The lancet Diabetes & endocrinology, 2018, 6(3): 237-48.DOI:10.1016/s2213-8587(17)30236-x

60. COLMAN E. Food and Drug Administration's Obesity Drug Guidance Document: a short history [J]. Circulation, 2012, 125(17): 2156-64.DOI:10.1161/circulationaha.111.028381

61. JASTREBOFF A M, KUSHNER R F. New Frontiers in Obesity Treatment: GLP-1 and Nascent Nutrient-Stimulated Hormone-Based Therapeutics [J]. Annual review of medicine, 2023, 74(125-39.DOI:10.1146/annurev-med-043021-014919

62. DEROSA G, MAFFIOLI P. Anti-obesity drugs: a review about their effects and their safety [J]. Expert opinion on drug safety, 2012, 11(3): 459-71.DOI:10.1517/14740338.2012.675326

63. HECK A M, YANOVSKI J A, CALIS K A. Orlistat, a new lipase inhibitor for the management of obesity [J]. Pharmacotherapy, 2000, 20(3): 270-9.DOI:10.1592/phco.20.4.270.34882

64. Chinese Diabetes Society, Chinese Medical Association. Guidelines for the Prevention and Treatment of Type 2 Diabetes in China (2020 Edition) [J]. Chinese Journal of Diabetes, 2021, 13(4): 315-409.DOI:10.3760/cma.j.cn115791-20210221-00095

65. SHI Q, WANG Y, HAO Q, et al. Pharmacotherapy for adults with overweight and obesity: a systematic review and network meta-analysis of randomised controlled trials [J]. Lancet (London, England), 2022, 399(10321): 259-69.DOI:10.1016/s0140-6736(21)01640-8

66. SMITH S R, STENLOF K S, GREENWAY F L, et al. Orlistat 60 mg reduces visceral adipose tissue: a 24-week randomized, placebo-controlled, multicenter trial [J]. Obesity (Silver Spring, Md), 2011, 19(9): 1796-803.DOI:10.1038/oby.2011.143

67. HENNESS S, PERRY C M. Orlistat: a review of its use in the management of obesity [J]. Drugs, 2006, 66(12): 1625-56.DOI:10.2165/00003495-200666120-00012

68. LUPIANEZ-MERLY C, DILMAGHANI S, VOSOUGHI K, et al. Review article: Pharmacologic management of obesity - updates on approved medications, indications and risks [J]. Alimentary pharmacology & therapeutics, 2024, 59(4): 475-91.DOI:10.1111/apt.17856

69. XU F, LIN B, ZHENG X, et al. GLP-1 receptor agonist promotes brown remodelling in mouse white adipose tissue through SIRT1 [J]. Diabetologia, 2016, 59(5): 1059-69.DOI:10.1007/s00125-016-3896-5

70. LóPEZ-FERRERAS L, RICHARD J E, NOBLE E E, et al. Lateral hypothalamic GLP-1 receptors are critical for the control of food reinforcement, ingestive behavior and body weight [J]. Molecular psychiatry, 2018, 23(5): 1157-68.DOI:10.1038/mp.2017.187

71. SECHER A, JELSING J, BAQUERO A F, et al. The arcuate nucleus mediates GLP-1 receptor agonist liraglutide-dependent weight loss [J]. The Journal of clinical investigation, 2014, 124(10): 4473-88.DOI:10.1172/jci75276

72. JELSING J, VRANG N, HANSEN G, et al. Liraglutide: short-lived effect on gastric emptying -- long lasting effects on body weight [J]. Diabetes Obes Metab, 2012, 14(6): 531-8.DOI:10.1111/j.1463-1326.2012.01557.x

73. CHEN K, CHEN L, SHAN Z, et al. Beinaglutide for weight management in Chinese individuals with overweight or obesity: A phase 3 randomized controlled clinical study [J]. Diabetes Obes Metab, 2024, 26(2): 690-8.DOI:10.1111/dom.15360

74. PI-SUNYER X, ASTRUP A, FUJIOKA K, et al. A Randomized, Controlled Trial of 3.0 mg of Liraglutide in Weight Management [J]. N Engl J Med, 2015, 373(1): 11-22.DOI:10.1056/NEJMoa1411892

75. DAVIES M J, BERGENSTAL R, BODE B, et al. Efficacy of Liraglutide for Weight Loss Among Patients With Type 2 Diabetes: The SCALE Diabetes Randomized Clinical Trial [J]. Jama, 2015, 314(7): 687-99.DOI:10.1001/jama.2015.9676

76. WILDING J P H, BATTERHAM R L, CALANNA S, et al. Once-Weekly Semaglutide in Adults with Overweight or Obesity [J]. N Engl J Med, 2021, 384(11): 989-1002.DOI:10.1056/NEJMoa2032183

77. DAVIES M, FæRCH L, JEPPESEN O K, et al. Semaglutide 2·4 mg once a week in adults with overweight or obesity, and type 2 diabetes (STEP 2): a randomised, double-blind, double-dummy, placebo-controlled, phase 3 trial [J]. Lancet (London, England), 2021, 397(10278): 971-84.DOI:10.1016/s0140-6736(21)00213-0

78. MU Y, BAO X, ELIASCHEWITZ F G, et al. Efficacy and safety of once weekly semaglutide 2·4 mg for weight management in a predominantly east Asian population with overweight or obesity (STEP 7): a double-blind, multicentre, randomised controlled trial [J]. The lancet Diabetes & endocrinology, 2024, 12(3): 184-95.DOI:10.1016/s2213-8587(23)00388-1

79. BUCKLEY S T, BæKDAL T A, VEGGE A, et al. Transcellular stomach absorption of a derivatized glucagon-like peptide-1 receptor agonist [J]. Science translational medicine, 2018, 10(467): 10.1126/scitranslmed.aar7047

80. KNOP F K, ARODA V R, DO VALE R D, et al. Oral semaglutide 50 mg taken once per day in adults with overweight or obesity (OASIS 1): a randomised, double-blind, placebo-controlled, phase 3 trial [J]. Lancet (London, England), 2023, 402(10403): 705-19.DOI:10.1016/s0140-6736(23)01185-6

81. KAWAI T, SUN B, YOSHINO H, et al. Structural basis for GLP-1 receptor activation by LY3502970, an orally active nonpeptide agonist [J]. Proceedings of the National Academy of Sciences of the United States of America, 2020, 117(47): 29959-67.DOI:10.1073/pnas.2014879117

82. WHARTON S, BLEVINS T, CONNERY L, et al. Daily Oral GLP-1 Receptor Agonist Orforglipron for Adults with Obesity [J]. N Engl J Med, 2023, 389(10): 877-88.DOI:10.1056/NEJMoa2302392

83. PRATT E, MA X, LIU R, et al. Orforglipron (LY3502970), a novel, oral non-peptide glucagon-like peptide-1 receptor agonist: A Phase 1b, multicentre, blinded, placebo-controlled, randomized, multiple-ascending-dose study in people with type 2 diabetes [J]. Diabetes Obes Metab, 2023, 25(9): 2642-9.DOI:10.1111/dom.15150

84. KARAGIANNIS T, AVGERINOS I, LIAKOS A, et al. Management of type 2 diabetes with the dual GIP/GLP-1 receptor agonist tirzepatide: a systematic review and meta-analysis [J]. Diabetologia, 2022, 65(8): 1251-61.DOI:10.1007/s00125-022-05715-4

85. JASTREBOFF A M, ARONNE L J, AHMAD N N, et al. Tirzepatide Once Weekly for the Treatment of Obesity [J]. N Engl J Med, 2022, 387(3): 205-16.DOI:10.1056/NEJMoa2206038

86. GARVEY W T, FRIAS J P, JASTREBOFF A M, et al. Tirzepatide once weekly for the treatment of obesity in people with type 2 diabetes (SURMOUNT-2): a double-blind, randomised, multicentre, placebo-controlled, phase 3 trial [J]. Lancet (London, England), 2023, 402(10402): 613-26.DOI:10.1016/s0140-6736(23)01200-x

87. ZHAO L, CHENG Z, LU Y, et al. Tirzepatide for Weight Reduction in Chinese Adults With Obesity: The SURMOUNT-CN Randomized Clinical Trial [J]. Jama, 2024, 10.1001/jama.2024.9217

88. LE ROUX C W, HANKOSKY E R, WANG D, et al. Tirzepatide 10 and 15 mg compared with semaglutide 2.4 mg for the treatment of obesity: An indirect treatment comparison [J]. Diabetes Obes Metab, 2023, 25(9): 2626-33.DOI:10.1111/dom.15148

89. CAPOZZI M E, D'ALESSIO D A, CAMPBELL J E. The past, present, and future physiology and pharmacology of glucagon [J]. Cell Metab, 2022, 34(11): 1654-74. DOI:10.1016/j.cmet.2022.10.001

90. JI L, JIANG H, LI H, et al. 1856-LB: Efficacy and Safety of Mazdutide in Chinese Participants with Overweight or Obesity (GLORY-1) [J]. Diabetes, 2024, 73(Supplement_1): 10.2337/db24-1856-LB

91. LE ROUX C W, STEEN O, LUCAS K J, et al. Glucagon and GLP-1 receptor dual agonist survodutide for obesity: a randomised, double-blind, placebo-controlled, dose-finding phase 2 trial [J]. The lancet Diabetes & endocrinology, 2024, 12(3): 162-73.DOI:10.1016/s2213-8587(23)00356-x

92. HAY D L, CHEN S, LUTZ T A, et al. Amylin: Pharmacology, Physiology, and Clinical Potential [J]. Pharmacological reviews, 2015, 67(3): 564-600.DOI:10.1124/pr.115.010629

93. FRIAS J P, DEENADAYALAN S, ERICHSEN L, et al. Efficacy and safety of co-administered once-weekly cagrilintide 2·4 mg with once-weekly semaglutide 2·4 mg in type 2 diabetes: a multicentre, randomised, double-blind, active-controlled, phase 2 trial [J]. Lancet (London, England), 2023, 402(10403): 720-30.DOI:10.1016/s0140-6736(23)01163-7

94. JASTREBOFF A M, KAPLAN L M, FRíAS J P, et al. Triple-Hormone-Receptor Agonist Retatrutide for Obesity - A Phase 2 Trial [J]. N Engl J Med, 2023, 389(6): 514-26.DOI:10.1056/NEJMoa2301972

95. FORZANO I, VARZIDEH F, AVVISATO R, et al. Tirzepatide: A Systematic Update [J]. International journal of molecular sciences, 2022, 23(23): 10.3390/ijms232314631

96. HANKOSKY E R, WANG H, NEFF L M, et al. Tirzepatide reduces the predicted risk of developing type 2 diabetes in people with obesity or overweight: Post hoc analysis of the SURMOUNT-1 trial [J]. Diabetes Obes Metab, 2023, 25(12): 3748-56.DOI:10.1111/dom.15269

97. LOOMBA R, HARTMAN M L, LAWITZ E J, et al. Tirzepatide for Metabolic Dysfunction-Associated Steatohepatitis with Liver Fibrosis [J]. N Engl J Med, 2024, 10.1056/NEJMoa2401943

98. NEWSOME P N, BUCHHOLTZ K, CUSI K, et al. A Placebo-Controlled Trial of Subcutaneous Semaglutide in Nonalcoholic Steatohepatitis [J]. N Engl J Med, 2021, 384(12): 1113-24.DOI:10.1056/NEJMoa2028395

99. JI L, JIANG H, ZHANG Y, et al. 1857-LB: Improvement of Liver Steatosis by Mazdutide in Chinese Participants with Overweight or Obesity—An Exploratory Analysis of GLORY-1 [J]. Diabetes, 2024, 73(Supplement_1): 10.2337/db24-1857-LB

100. MALHOTRA A, GRUNSTEIN R R, FIETZE I, et al. Tirzepatide for the Treatment of Obstructive Sleep Apnea and Obesity [J]. N Engl J Med, 2024, 10.1056/NEJMoa2404881

101. LINCOFF A M, BROWN-FRANDSEN K, COLHOUN H M, et al. Semaglutide and Cardiovascular Outcomes in Obesity without Diabetes [J]. N Engl J Med, 2023, 389(24): 2221-32.DOI:10.1056/NEJMoa2307563

102. HANKOSKY E R, WANG H, NEFF L M, et al. Tirzepatide reduces the predicted risk of atherosclerotic cardiovascular disease and improves cardiometabolic risk factors in adults with obesity or overweight: SURMOUNT-1 post hoc analysis [J]. Diabetes Obes Metab, 2024, 26(1): 319-28.DOI:10.1111/dom.15318

103. HEERSPINK H J, FRIEDMAN A N, BJORNSTAD P, et al. Effect of Tirzepatide on Kidney Function in People with Excess Body Weight: A Post Hoc Analysis of the SURMOUNT-1 Trial: FR-OR47 [J]. 2023, 34(11S): 41.DOI:10.1681/ASN.20233411S141b

104. BJERRE KNUDSEN L, MADSEN L W, ANDERSEN S, et al. Glucagon-like Peptide-1 receptor agonists activate rodent thyroid C-cells causing calcitonin release and C-cell proliferation [J]. Endocrinology, 2010, 151(4): 1473-86.DOI:10.1210/en.2009-1272

105. LISCO G, DE TULLIO A, DISOTEO O, et al. Glucagon-like peptide 1 receptor agonists and thyroid cancer: is it the time to be concerned? [J]. Endocrine connections, 2023, 12(11): 10.1530/ec-23-0257

106. ELASHOFF M, MATVEYENKO A V, GIER B, et al. Pancreatitis, pancreatic, and thyroid cancer with glucagon-like peptide-1-based therapies [J]. Gastroenterology, 2011, 141(1): 150-6. DOI:10.1053/j.gastro.2011.02.018

107. VAN BAAK M A, MARIMAN E C M. Mechanisms of weight regain after weight loss - the role of adipose tissue [J]. Nature reviews Endocrinology, 2019, 15(5): 274-87.DOI:10.1038/s41574-018-0148-4

108. CORNIER M A. Is your brain to blame for weight regain? [J]. Physiology & behavior, 2011, 104(4): 608-12. DOI:10.1016/j.physbeh.2011.04.003

109. WILDING J P H, BATTERHAM R L, DAVIES M, et al. Weight regain and cardiometabolic effects after withdrawal of semaglutide: The STEP 1 trial extension [J]. Diabetes Obes Metab, 2022, 24(8): 1553-64.DOI:10.1111/dom.14725

110. RUBINO D, ABRAHAMSSON N, DAVIES M, et al. Effect of Continued Weekly Subcutaneous Semaglutide vs Placebo on Weight Loss Maintenance in Adults With Overweight or Obesity: The STEP 4 Randomized Clinical Trial [J]. JAMA, 2021, 325(14): 1414-25.DOI:10.1001/jama.2021.3224

111. GARVEY W T, MECHANICK J I, BRETT E M, et al. AMERICAN ASSOCIATION OF CLINICAL ENDOCRINOLOGISTS AND AMERICAN COLLEGE OF ENDOCRINOLOGY COMPREHENSIVE CLINICAL PRACTICE GUIDELINES FOR MEDICAL CARE OF PATIENTS WITH OBESITY [J]. Endocrine practice: official journal of the American College of Endocrinology and the American Association of Clinical Endocrinologists, 2016, 22 Suppl 3(1-203.DOI:10.4158/ep161365.Gl

112. WANG L, GAO P, ZHANG M, et al. Prevalence and Ethnic Pattern of Diabetes and Prediabetes in China in 2013 [J]. JAMA, 2017, 317(24): 2515-23.DOI:10.1001/jama.2017.7596

113. HAO Q, AERTGEERTS B, GUYATT G, et al. PCSK9 inhibitors and ezetimibe for the reduction of cardiovascular events: a clinical practice guideline with risk-stratified recommendations [J]. BMJ (Clinical research ed), 2022, 377(e069066.DOI:10.1136/bmj-2021-069066

114. DING S, GUO LX. Research Progress on Obesity and Obstructive Sleep Apnea Syndrome [J]. Journal of Clinical Internal Medicine, 2020, 37(9): 616-9. DOI:10.3969/j.issn.1001⁃9057.2020.09.003

115. LIANG P, XI L, SHI J, et al. Prevalence of polycystic ovary syndrome in Chinese obese women of reproductive age with or without metabolic syndrome [J]. Fertility and sterility, 2017, 107(4): 1048-54. DOI:10.1016/j.fertnstert.2016.12.029

116. KHAN S S, NING H, WILKINS J T, et al. Association of Body Mass Index With Lifetime Risk of Cardiovascular Disease and Compression of Morbidity [J]. JAMA cardiology, 2018, 3(4): 280-7.DOI:10.1001/jamacardio.2018.0022

117. GONG M, WEN S, NGUYEN T, et al. Converging Relationships of Obesity and Hyperuricemia with Special Reference to Metabolic Disorders and Plausible Therapeutic Implications [J]. Diabetes, metabolic syndrome and obesity: targets and therapy, 2020, 13(943-62.DOI:10.2147/dmso.S232377

118. DEHLIN M, JACOBSSON L, RODDY E. Global epidemiology of gout: prevalence, incidence, treatment patterns and risk factors [J]. Nature reviews Rheumatology, 2020, 16(7): 380-90.DOI:10.1038/s41584-020-0441-1

119. JIANG Z, WANG Y, ZHAO X, et al. Obesity and chronic kidney disease [J]. American journal of physiology Endocrinology and metabolism, 2023, 324(1): E24-e41.DOI:10.1152/ajpendo.00179.2022

120. QIAN J, LI N, REN X. Obesity and depressive symptoms among Chinese people aged 45 and over [J]. Sci Rep, 2017, 7(45637.DOI:10.1038/srep45637

121. SILVERII G A, MARINELLI C, MANNUCCI E, et al. Glucagon-like peptide-1 receptor agonists and mental health: A meta-analysis of randomized controlled trials [J]. Diabetes Obes Metab, 2024, 10.1111/dom.15538

122. ALONSO-PEDRERO L, BES-RASTROLLO M, MARTI A. Effects of antidepressant and antipsychotic use on weight gain: A systematic review [J]. Obes Rev, 2019, 20(12): 1680-90.DOI:10.1111/obr.12934

123. ZHANG L, CHEN J, ZHANG J, et al. Regional Disparities in Obesity Among a Heterogeneous Population of Chinese Children and Adolescents [J]. JAMA network open, 2021, 4(10): e2131040.DOI:10.1001/jamanetworkopen.2021.31040

124. Hills AP, Andersen LB, Byrne NM. Physical activity and obesity in children. British Journal of Sports Medicine, 2011, 45(11), 866-870. doi:10.1136/bjsports-2011-090199.

125. LANIGAN J, BARBER S, SINGHAL A. Prevention of obesity in preschool children [J]. The Proceedings of the Nutrition Society, 2010, 69(2): 204-10.DOI:10.1017/s0029665110000029

126. REINEHR T, WABITSCH M. Childhood obesity [J]. Current opinion in lipidology, 2011, 22(1): 21-5.DOI:10.1097/MOL.0b013e32833f9c37

127. HINNEY A, KöRNER A, FISCHER-POSOVSZKY P. The promise of new anti-obesity therapies arising from knowledge of genetic obesity traits [J]. Nature reviews Endocrinology, 2022, 18(10): 623-37.DOI:10.1038/s41574-022-00716-0

128. KAPOOR E, FAUBION S S, KLING J M. Obesity Update in Women [J]. Journal of women's health (2002), 2019, 28(12): 1601-5.DOI:10.1089/jwh.2019.8041

129. Tan HS, Habib AS. Obesity in women: Anaesthetic implications for peri-operative and peri-partum management. Anaesthesia, 2021, 76(Suppl 4), 108-117. doi:10.1111/anae.15403.

130. HAQQ A M, CHUNG W K, DOLLFUS H, et al. Efficacy and safety of setmelanotide, a melanocortin-4 receptor agonist, in patients with Bardet-Biedl syndrome and Alström syndrome: a multicentre, randomised, double-blind, placebo-controlled, phase 3 trial with an open-label period [J]. The lancet Diabetes & endocrinology, 2022, 10(12): 859-68.DOI:10.1016/s2213-8587(22)00277-7

131. BATSIS J A, VILLAREAL D T. Sarcopenic obesity in older adults: aetiology, epidemiology and treatment strategies [J]. Nature reviews Endocrinology, 2018, 14(9): 513-37.DOI:10.1038/s41574-018-0062-9

132. JOSHI G P. Anesthetic Considerations in Adult Patients on Glucagon-Like Peptide-1 Receptor Agonists: Gastrointestinal Focus [J]. Anesthesia and analgesia, 2024, 138(1): 216-20.DOI:10.1213/ane.0000000000006810

133. BUSETTO L, DICKER D, AZRAN C, et al. Practical Recommendations of the Obesity Management Task Force of the European Association for the Study of Obesity for the Post-Bariatric Surgery Medical Management [J]. Obesity facts, 2017, 10(6): 597-632.DOI:10.1159/000481825

134. LUCAS E, SIMMONS O, TCHANG B, et al. Pharmacologic management of weight regain following bariatric surgery [J]. Frontiers in endocrinology, 2022, 13(1043595.DOI:10.3389/fendo.2022.1043595

135. REDMOND I P, SHUKLA A P, ARONNE L J. Use of Weight Loss Medications in Patients after Bariatric Surgery [J]. Current obesity reports, 2021, 10(2): 81-9.DOI:10.1007/s13679-021-00425-1

136. SCHWARTZ J, CHAUDHRY U I, SUZO A, et al. Pharmacotherapy in Conjunction with a Diet and Exercise Program for the Treatment of Weight Recidivism or Weight Loss Plateau Post-bariatric Surgery: a Retrospective Review [J]. Obesity surgery, 2016, 26(2): 452-8.DOI:10.1007/s11695-015-1979-x

137. STANFORD F C, ALFARIS N, GOMEZ G, et al. The utility of weight loss medications after bariatric surgery for weight regain or inadequate weight loss: A multi-center study [J]. Surgery for obesity and related diseases: official journal of the American Society for Bariatric Surgery, 2017, 13(3): 491-500. DOI:10.1016/j.soard.2016.10.018

138. PAJECKI D, HALPERN A, CERCATO C, et al. Short-term use of liraglutide in the management of patients with weight regain after bariatric surgery [J]. Revista do Colegio Brasileiro de Cirurgioes, 2013, 40(3): 191-5.DOI:10.1590/s0100-69912013000300005

139. WEBB D L, ABRAHAMSSON N, SUNDBOM M, et al. Bariatric surgery - time to replace with GLP-1? [J]. Scandinavian journal of gastroenterology, 2017, 52(6-7): 635-40.DOI:10.1080/00365521.2017.1293154

140. MAHMOUD R, KIMONIS V, BUTLER M G. Genetics of Obesity in Humans: A Clinical Review [J]. International journal of molecular sciences, 2022, 23(19): 10.3390/ijms231911005

141. MARKHAM A. Setmelanotide: First Approval [J]. Drugs, 2021, 81(3): 397-403.DOI:10.1007/s40265-021-01470-9

142. CHOU K, PERRY C M. Metreleptin: first global approval [J]. Drugs, 2013, 73(9): 989-97.DOI:10.1007/s40265-013-0074-7
